# Shotgun Metagenomics of Gastric Biopsies Reveals Compositional and Functional Microbiome Shifts in High- and Low-Gastric-Cancer-Risk Populations from Colombia, South America

**DOI:** 10.1080/19490976.2023.2186677

**Published:** 2023-03-12

**Authors:** Anthony Mannion, Alexander Sheh, Zeli Shen, JoAnn Dzink-Fox, M. Blanca Piazuelo, Keith T Wilson, Richard Peek, James G. Fox

**Affiliations:** a Division of Comparative Medicine, Massachusetts Institute of Technology, Cambridge, Massachusetts, USA; b Department of Medicine, Vanderbilt University Medical Center, Nashville, Tennessee, USA; c Department of Biological Engineering, Massachusetts Institute of Technology, Cambridge, Massachusetts, USA

**Keywords:** *Helicobacter pylori*, gastric cancer, gastric microbiome, whole metagenomic shotgun sequencing, 16S rRNA sequencing

## Abstract

Along with *Helicobacter pylori* infection, the gastric microbiota is hypothesized to modulate stomach cancer risk in susceptible individuals. Whole metagenomic shotgun sequencing (WMS) is a sequencing approach to characterize the microbiome with advantages over traditional culture and 16S rRNA sequencing including identification of bacterial and non-bacterial taxa, species/strain resolution, and functional characterization of the microbiota. In this study, we used WMS to survey the microbiome in extracted DNA from antral gastric biopsy samples from Colombian patients residing in the high-risk gastric cancer town Túquerres (*n* = 10, *H. pylori*-positive = 7) and low-risk town of Tumaco (*n* = 10, *H. pylori*-positive = 6). Kraken2/Bracken was used for taxonomic classification and abundance. Functional gene profiles were inferred by InterProScan and KEGG analysis of assembled contigs and gene annotation. The most abundant taxa represented bacteria, non-human eukaryota, and viral genera found in skin, oral, food, and plant/soil environments including *Staphylococus*, *Streptococcus*, *Bacillus*, *Aspergillus*, and *Siphoviridae*. *H. pylori* was the predominant taxa present in *H. pylori*-positive samples. Beta diversity was significantly different based on *H. pylori*-status, risk group, and sex. WMS detected more bacterial taxa than 16S rRNA sequencing and aerobic, anaerobic, and microaerobic culture performed on the same gastric biopsy samples. WMS identified significant differences in functional profiles found between *H. pylori*-status, but not risk or sex groups. *H. pylori*-positive samples were significantly enriched for *H. pylori*-specific genes including virulence factors such as *vacA*, *cagA*, and urease, while carbohydrate and amino acid metabolism genes were enriched in *H. pylori*-negative samples. This study shows WMS has the potential to characterize the taxonomy and function of the gastric microbiome as risk factors for *H. pylori*-associated gastric disease. Future studies will be needed to compare and validate WMS versus traditional culture and 16S rRNA sequencing approaches for characterization of the gastric microbiome.

## Introduction

Although *Helicobacter pylori* infection is the strongest factor associated with developing gastritis and stomach cancer, increasing evidence suggests the gastric microbiota may also have a key role in modulating disease risk in susceptible individuals^1–3^. Culture and 16S rRNA sequencing have been the primary means to study the gastric microbiome, and while these approaches have revealed important insights into the potential influence of the microbiome on gastric disease risk and progression, these methods have limited scope including low-throughput or nonspecific taxonomic identification. Unlike 16S rRNA sequencing, metagenomic shotgun sequencing (WMS) is an untargeted, microbiome sequencing approach that enables 1) identification of bacterial, eukaryota, viral, and archaea microbes, 2) species and strain level taxonomic resolution, and 3) functional characterization of the microbiota^4,5^. As a result, WMS is being increasingly used with 16S rRNA profiling to study the gastrointestinal microbiome, especially to identify risk factors associated with intestinal inflammatory diseases and cancers^6–12^. While numerous studies have characterized the gastric microbiome using 16S rRNA profiling^1^, WMS provides an opportunity to further define how the microbiome taxonomic composition and function may contribute as factors associated with disease risk.

In this study, we utilized WMS to survey the microbiome in gastric biopsy samples collected from Colombian patients residing in the high gastric cancer risk (HGCR) town of Túquerres located in the Andes Mountains and low gastric cancer risk (LGCR) coastal town of Tumaco. While both populations have high prevalence of *H. pylori* infection (>80%), additional factors have been extensively investigated over the last four decades to explain the differences in gastric cancer risk. Geographic location has been hypothesized to represent multifactorial influences of host and *H. pylori* genotypes as well as dietary and environmental variables including helminth infection and host microbiome composition/function^13^. Previous investigations of these Colombian cohorts by traditional culture and 16S rRNA profiling have revealed significant differences in the gastric microbiome composition between gastric cancer populations^13–15^. Thus, in the current study, the gastric microbiome was further evaluated using WMS to assess its association as a risk factor for disease in these patient cohorts.

A significant challenge for next-generation sequenced-based profiling of the microbiome in tissue biopsy samples is the low abundance of microbe to host cells, especially in the stomach where microbial density is 5–10 log-folds lower compared to the lower intestine and feces^16^. 16S rRNA profiling attempts to overcome this challenge via PCR amplification of 16S rRNA genes regions prior to sequencing. However, this method is prone to technical artifacts due to amplification bias and sensitivity to contamination^17,18^. Conversely, WMS does not require PCR and therefore potentially provides a more accurate reflection of the gut microbiota compared to 16S rRNA profiling^11^. Despite this advantage, WMS is substantially more expensive and resource intensive than 16S rRNA profiling because magnitudes of deeper sequencing are required to detect microbiota genes in tissue samples with low microbe-to-human DNA ratios, even with methods to deplete host DNA prior to sequencing^19^. Thus, an objective of the current study was to compare gastric microbiota profiles between HGCR and LGCR tissue samples using WMS versus 16S rRNA approaches as well as by traditional bacterial culture. To our knowledge, this study is the first to use WMS, 16S rRNA and culturing profiling methodologies to evaluate and compare the gastric microbiome. We hypothesize WMS is a feasible approach to characterize the composition and functional potential of the gastric microbiota from human stomach biopsy tissue as well as identify significant features that differentiate patients from HGCR and LGCR populations.

## Results

### Study population

Twenty gastric biopsy tissue samples for microbiome analysis were selected to match for *H. pylori* status, risk group, histopathology scores, age, and sex from a total set of 163 adult patients undergoing upper gastrointestinal endoscopy from the HGCR and LGCR in Colombia (Table 1). There were no statistical differences in age between *H. pylori* status, risk, or sex groups. Histopathology scores were not statistically different based on *H. pylori* status or risk group, but samples from males had significantly higher pathology scores compared to those from females (*P* = 0.008, Mann – Whitney U-test).

**Table 1.** *H. pylori* status, risk group, histopathology scores, age, and sex of patient gastric biopsy samples from low and high gastric cancer risk populations in Colombia.

Sample ID	Subject ID	Town	Risk	Sex	Age	Histological Diagnosis^a^	Histology Score	*H. pylori* status^b^
E1	MT5168	Tumaco	Low Risk	Female	41	NAG	1.33	Negative
A5	MT5170	Tumaco	Low Risk	Female	59	NAG	1.33	Negative
H4	MT5122	Tumaco	Low Risk	Female	47	NAG	1.67	Positive
F2	MT5127	Tumaco	Low Risk	Female	48	NAG	2.33	Positive
C5	MT5125	Tumaco	Low Risk	Female	53	NAG	2.67	Positive
F3	MT5104	Tumaco	Low Risk	Male	53	MAG-IM	4.7	Negative
C2	MT5175	Tumaco	Low Risk	Male	59	NAG	2.33	Positive
B4	MT5156	Tumaco	Low Risk	Male	51	NAG	2.67	Positive
D5	MT5178	Tumaco	Low Risk	Male	50	NAG	3	Positive
B5	MT5179	Tumaco	Low Risk	Male	52	NAG	3	Positive
G1	MT2181	Túquerres	High Risk	Female	46	NAG	1.33	Negative
H2	MT2162	Túquerres	High Risk	Female	41	NAG	1.67	Negative
E5	MT2176	Túquerres	High Risk	Female	52	NAG	2	Positive
E2	MT2172	Túquerres	High Risk	Female	45	NAG	2.33	Positive
D3	MT2164	Túquerres	High Risk	Female	40	NAG	2.67	Positive
A4	MT2126	Túquerres	High Risk	Male	46	MAG-IM	4.6	Negative
C4	MT2143	Túquerres	High Risk	Male	57	MAG-IM	4.3	Positive
G2	MT2116	Túquerres	High Risk	Male	57	NAG	1.33	Negative
E4	MT2147	Túquerres	High Risk	Male	41	NAG	2.67	Positive
D4	MT2141	Túquerres	High Risk	Male	45	NAG	2.67	Positive

^a^NAG, non-atrophic gastritis; MAG-IM, multifocal atrophic gastritis with intestinal metaplasia.

^b^*H.* *pylori* status determined by a modified Steiner stain and culture.

### WMS taxonomic profiling of the gastric microbiome

WMS yielded 35,062,050 to 168,230,930 total reads per sample after quality control. Of these reads, WMS profiling classified ~96–99% reads as host (i.e., *Homo sapiens*), and ~1–4% reads were unclassified taxa (supplemental figure S1). The remaining reads belonged to bacteria (~0.03–0.2%), archaea (~0.0007–0.002%), virus (~0.002–0.006%), and non-human eukaryota (~0.03–0.1%) taxa (supplemental figure S1). Thus, 42,319 to 304,298 total reads per sample classified as bacteria, non-human eukaryota, viral, and archaea operational taxonomic units (OTUs) were further evaluated in this study (supplemental figure S1). There were no statistical differences in the number of reads in total or per taxa between *H. pylori* status, risk group, or sexes, except that males had significantly higher bacterial reads than females (86,045.0 ± 38,847.2 vs. 51,290.7 ± 26,999.1, *P* = 0.03). Of these, bacteria and non-human eukaryotes predominated. Virus followed by archaea were the next most abundant. At the bacterial genus level, WMS identified significantly more OTUs compare to 16S rRNA sequencing (*P* < 0.001) (supplemental figure S2).

**Figure 1.**
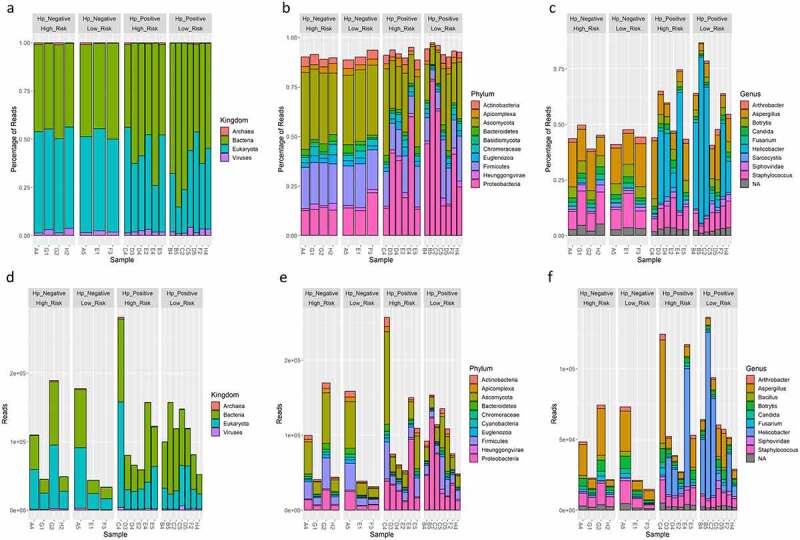
Relative percent of reads classified as bacteria, archaea, virus, and non-human eukaryota OTUs at the kingdom (A), phylum (B), and genus (C) level using WMS. Absolute reads classified as bacteria, archaea, virus, and non-human eukaryota OTUs at the kingdom (D), phylum (E), and genus (F) level using WMS. The top 10 most abundant OTUs are shown.

**Figure 2.**
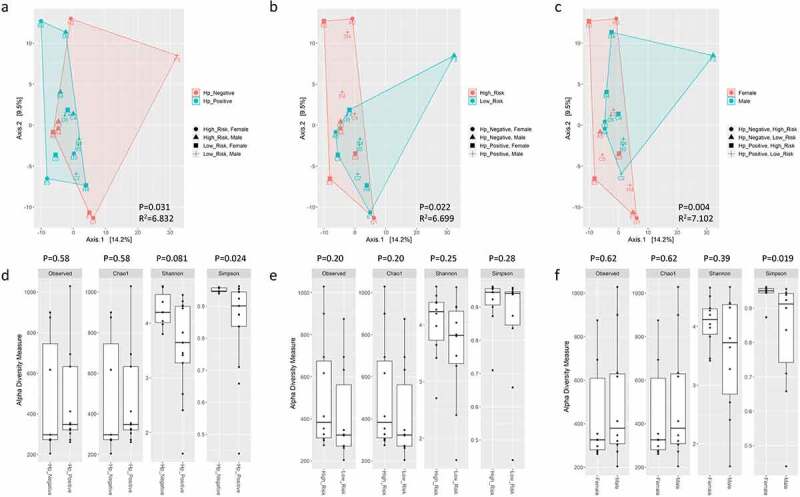
PCA plots of Aitchison distance for beta diversity comparisons of bacteria, archaea, virus, and non-human eukaryota OTUs at the genus level detected using WMS for *H. pylori* status (A), risk group (B), and sex (C) samples. Statistical analysis for beta diversity was performed using PERMANOVA to determine significance differences (P-value) and percentage of the variance explained (R^2^) the between groups. Observed OTUs, Chao1, Shannon and Simpson alpha diversity metrics on bacteria, archaea, virus, and non-human eukaryota OTUs at the genus level detected using WMS for *H. pylori* status (D), risk group (E), and sex (F) samples. Statistical comparisons of alpha diversity metrics between groups was performed using the Wilcoxon rank sum test with P-value adjustment using the Holm method.

When all taxa (i.e., bacteria, archaea, virus, and non-human eukaryota) were evaluated, the top 10 most abundant OTUs represented bacteria, non-human eukaryota, and viral genera that have been described in skin, oral, small/large intestine, food, plant, and soil environments (Figure 1). Beta diversity was significantly different based on *H. pylori* status (*P* = 0.031), risk group (*P* = 0.022), and sex (*P* = 0.004) (Figure 2). Observed OTUs, Chao1, and Shannon alpha diversity metrics were not significant between these groups. The Simpson alpha diversity metric was significantly lower in *H. pylori*-positive samples (*P* = 0.024) and males (*P* = 0.019) (Figure 2). When Helicobacter reads were excluded from these analyses, beta diversity was no longer statistically different between *H. pylori*-positive versus *H. pylori*-negative samples, but remained significantly different between risk groups and sexes (supplemental figure S3). There was no significance difference in alpha diversity metrics when Helicobacter reads were excluded from analyses (supplemental figure S3). Differential abundance analysis by GLM and T-test identified that males were significantly enriched in *Keratinibaculum* spp., regardless if *H. pylori* reads were included or excluded (supplemental figure S5A). No OTUs were statistically abundant between the *H. pylori* status or risk groups. While sample F3 appeared to be an outlier in the PCA plots, statistical significance for beta diversity did not change when omitted, suggesting this sample did not skew results (results not shown).

**Figure 3.**
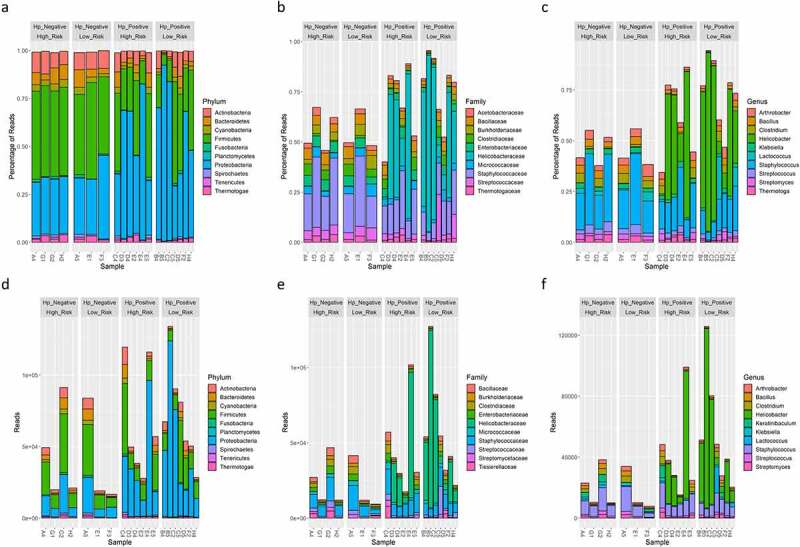
Relative percent of reads classified as bacteria OTUs at the phylum(A), family(B), and genus (C) level using WMS. Absolute reads classified as bacteria OTUs at the phylum(E), family(F), and genus (G) level using WMS. The top 10 most abundant OTUs are shown.

**Figure 4.**
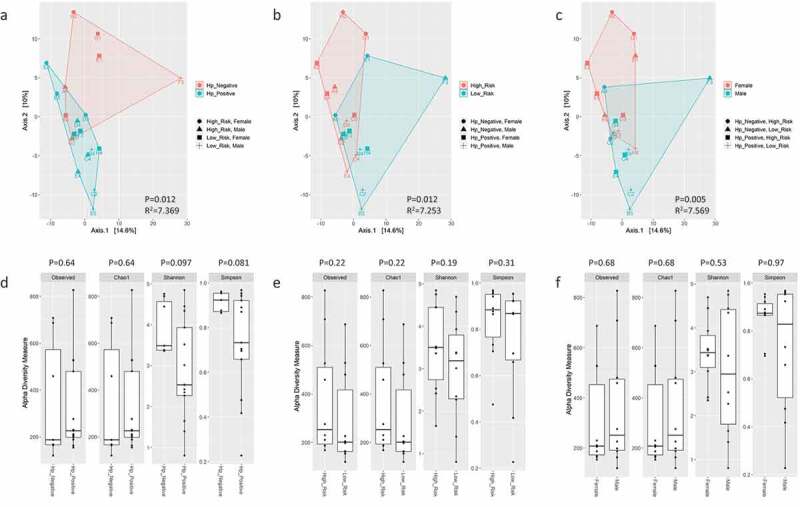
PCA plots of Aitchison distance for beta diversity comparisons of bacteria OTUs at the genus level detected using WMS between *H. pylori* status (A), risk group (B), and sex (C) samples. Statistical analysis for beta diversity was performed using PERMANOVA to determine significance differences (P-value) and percentage of the variance explained (R^2^) between the groups. Observed OTUs, Chao1, Shannon and Simpson alpha diversity metrics on bacteria OTUs at the genus level detected using WMS for *H. pylori* status (D), risk group (E), and sex (F) samples. Statistical comparisons of alpha diversity metrics between groups was performed using the Wilcoxon rank sum test with P-value adjustment using the Holm method.

Analyses repeated on only bacteria taxa (i.e., archaea, virus, and non-human eukaryote taxa excluded) identified that genera associated with the skin, oral, small/large intestine, and soil were the most abundant in samples (Figure 3) and predominately belonged to the *Proteobacteria* and *Firmicutes* phyla. *Helicobacter* and *Klebsiella* spp. were the most abundant *Proteobacteria* detected. *Staphylococcus*, *Streptococcus*, *Bacillus*, and *Lactococcus*, and *Clostridium* spp., all of which were *Firmicutes*, were within the 10 most abundant bacterial taxa detected. The soil-associated genera *Arthrobacter* and *Thermatoga* spp. were also present. A significant difference in beta diversity was again present based on *H. pylori* status (*P* = 0.012), risk group (*P* = 0.012), and sex (*P* = 0.005) (Figure 4). There were no significant differences in alpha diversity metrics based on *H. pylori* status when Helicobacter reads were included or excluded from analyses (Figure 4, supplemental figure S4). If Helicobacter reads were excluded, beta diversity remained significantly different between risk groups (*P* = 0.011) and sex (*P* = 0.049) (supplemental figure S4). *Keratinibaculum* spp. remained significantly enriched in males when only bacterial taxa were evaluated (supplemental figure S5B). Statistical significance for beta diversity did not change when sample F3 was omitted from PCA plots (results not shown).

There was no significant difference in alpha or beta diversity between groups when non-human eukaryote (supplemental figure S6), archaea (supplemental figure S7), or viral (supplemental figure S8) taxa were individually analyzed. *Aspergillus* spp., a fungal genus, was the predominant non-human eukaryote OTU noted (supplemental figure S9). *Siphoviridae* spp., which target bacterial and archaea hosts, were the most enriched viral OTU in all samples (supplemental figure S10). Of archaea, *Pyrolobus* species were the most common (supplemental figure S11). Together, these results indicate that *H. pylori* alters the structure and diversity of the gastric microbiome and primarily affects bacterial communities. Non-bacterial taxa were in general uniform across samples, regardless of *H. pylori* infection status. Since significant differences in diversity were not present for other taxonomic kingdoms, bacteria appear to be the primary constituents of gastric microbiota diversity.

### 16S rRNA versus WMS for bacterial microbiome characterization

**Figure 5.**
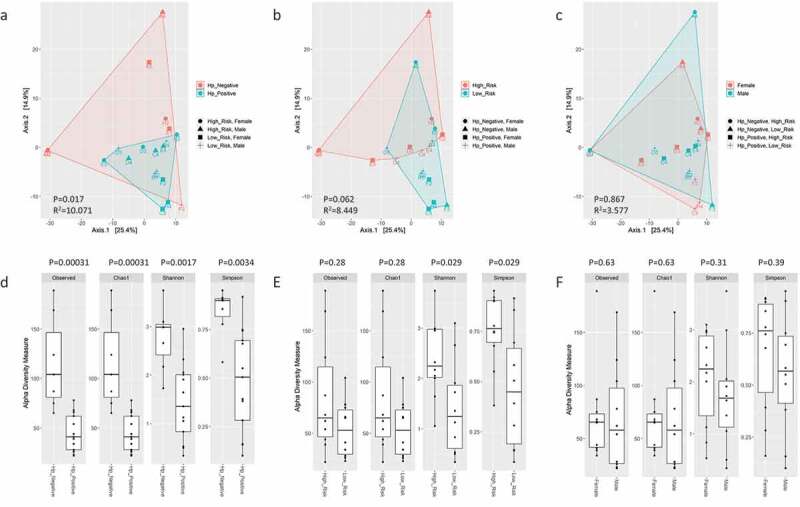
PCA plots of Aitchison distance for beta diversity comparisons of bacteria OTUs at the genus level detected using 16S rRNA profiling for *H. pylori* status (A), risk group (B), and sex (C) samples. Statistical analysis for beta diversity was performed using PERMANOVA to determine significance differences (P-value) and percentage of the variance explained (R^2^) between the groups. Observed OTUs, Chao1, Shannon and Simpson alpha diversity metrics of bacteria OTUs at the genus level detected using 16S rRNA profiling for *H. pylori* status (D), risk group (E), and sex (F). Statistical comparisons of alpha diversity metrics between groups was performed using the Wilcoxon rank sum test with P-value adjustment using the Holm method.

**Figure 6.**
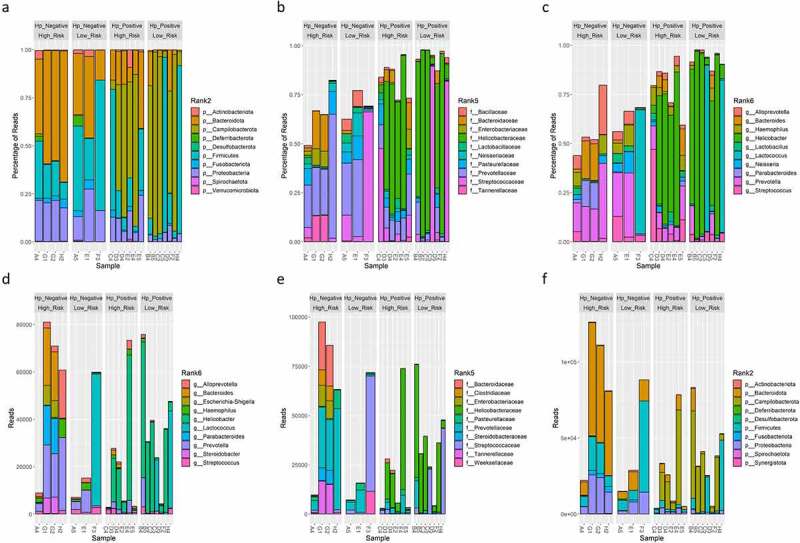
Relative percent of reads classified as bacterial OTUs at the phylum (A), family (B), and genus (C) level detected using 16S rRNA profiling. Absolute reads classified as bacterial OTUs at the phylum (D), family (E), and genus (F) level detected using 16S rRNA profiling. The top 10 most abundant OTUs are shown.

Unlike WMS analysis, 16S rRNA profiling only identified significant differences in beta diversity based on *H. pylori*-status (Figure 5). Additionally, observed OTUs, Chao1, Shannon, and Simpson indices were significantly lower in *H. pylori*-positive samples (Figure 5). These alpha diversity metrics suggests *H. pylori* outcompetes other microbes for colonization in the gastric niche, and when *H. pylori* is absent, the gastric niche may be occupied by a higher diversity of microbes. When Helicobacter reads were excluded, beta diversity as well as Shannon and Simpson indices were no longer statistically different between *H. pylori*-status groups (supplemental figure 12). Only *Helicobacter*, *Streptococcus*, and *Lactococcus* were shared between the top 10 most abundant bacterial genera between 16S rRNA versus WMS profiling (Figure 6). Linear regression of relative abundance at the genus level was performed to evaluate how similar the microbiome profiles were between 16S rRNA and WMS per sample. Seven of thirteen *H. pylori*-positive samples had strong coefficient of determinations (R^2^>0.75), suggesting these 16S rRNA and WMS methods yielded similar gastric microbiome profiles (supplemental figure 13A). Interestingly, when Helicobacter reads were removed from the linear regression analysis, these R^2^ values became less than 0.1 (supplemental figure 13B). For the remaining 5 *H. pylori*-positive and 7 *H. pylori*-negative samples, microbiome profiles at the genus level were poorly correlated between 16S rRNA and WMS methods, as shown by low R^2^ values (supplemental figure 13A). These findings indicate that gastric microbiome profiles drastically differ based on the sequencing methodology utilized.

### Culture versus 16S rRNA and WMS for bacterial microbiome characterization

**Figure 7.**
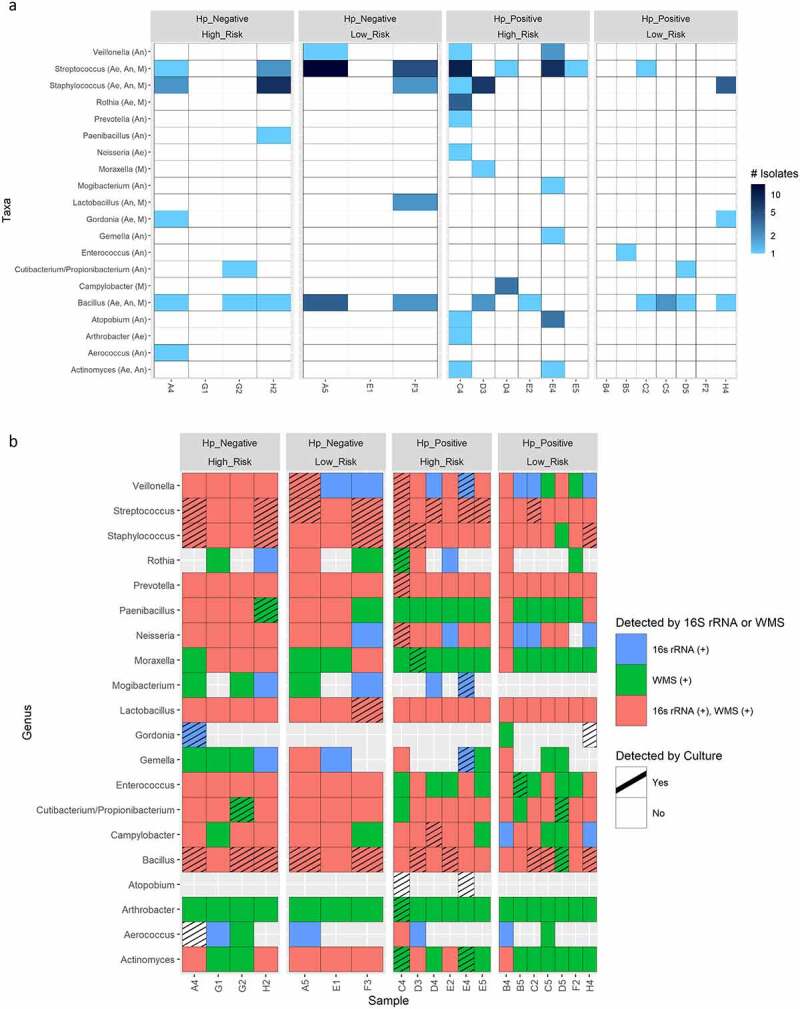
A) Heatmap showing absolute number non-Helicobacter bacterial isolates cultured per genera from each gastric biopsy sample. Culture conditions in which genera were isolated are indicated in parenthesis next to taxa names. Ae, aerobic; An, anaerobic; M, microaerobic. B) The concurrence of genera detected by culture, 16s rRNA and/or WMS profiling per gastric biopsy sample using a heatmap showing the binary presence/absence of genera per method. Note hashed boxes represent the 49 total instances in which at least one isolate from specific genus was cultured from a gastric biopsy sample.

Bacterial genera detected by 16S rRNA and WMS analysis were compared with those cultured from gastric biopsies. 117 non-Helicobacter bacteria isolates representing 20 genera were cultured from 17 gastric biopsy samples under aerobic, anaerobic, and microaerobic conditions (Figure 7A). No non-Helicobacter bacteria were cultured in three samples (B4, E1, and G1; Figure 7A). Non-Helicobacter bacterial genera identified by 16S rRNA, WMS, and culture profiling were plotted on a binary presence/absence heatmap to determine the concurrence of detection between methods per sample (Figure 7B). For culture-based detection, there were 49 instances in which a specific genus was present in a specific gastric biopsy sample (hashed boxes in Figure 7B). WMS had more of these genera in common with culture than 16S rRNA sequencing (41/49 versus 35/49, respectively) (Figure 7B), but these proportions were not statistically different using the z-score test. Thirty-one of the isolates detected by culture were identified by both WMS and 16S rRNA profiling (Figure 7B). Interestingly, culture detected four isolates not identified by 16S rRNA or WMS profiling (*Gordonia* in sample H4, *Atopobium* in samples C4 and E4, and *Aerococcus* in sample A4). While *Gordonia* and *Aerococcus* were identified by 16S rRNA or WMS profiling in other samples (*Gordonia* in samples A4 and B4, *Aerococcus* G1, G2, A5, C4, D3, B4, C5), *Atopobium* was not detected by either sequencing approach in any of the 20 biopsies (Figure 7B). Under the aerobic, anaerobic, and microaerobic conditions, growth for fungi or archaea were not observed after 2–3 weeks of culture from any gastric biopsy sample.

### Functional prediction of bacterial gastric microbiome from WMS

WMS reads classified as bacterial were assembled into contigs and annotated for protein coding genes. Bacterial reads were assembled into 1,361 to 7,628 contigs comprising 289,174 to 2,366,126 bp of total metagenomics sequence and included 1,120 to 5,513 annotated protein coding genes (supplemental table S1). DIAMOND analysis of the annotated protein gene sequences against the nr database was performed to determine the closest taxonomic homolog for each gene. Beta diversity in the taxonomic profiles of annotated protein genes was statistically different between *H. pylori*-positive versus *H. pylori*-negative samples (supplemental figure S14). Only genes corresponding to *Helicobacter* spp. were statistically more abundant in *H. pylori*-positive compared to *H. pylori*-negative samples based on differential abundance analysis by GLM and T-test (FDR<0.001). There were no statistical differences in beta diversity or differentially abundant genes between risk groups or sexes (supplemental figure 14). *H. pylori*-positive compared to *H. pylori*-negative samples were assembled into more contigs (3,565.4 ± 1,983.1 vs. 3,941.0 ± 1,474.6, *P* = 0.635) with larger overall metagenomics sequence (740,570.4 ± 379,153.0 vs. 1,590,043.4 ± 536,639.7, *P* = 0.002) as well as had more genes annotated (2,435.6 ± 1,038.4 vs. 3,812.1 ± 885.3, *P* = 0.006).

**Figure 8.**
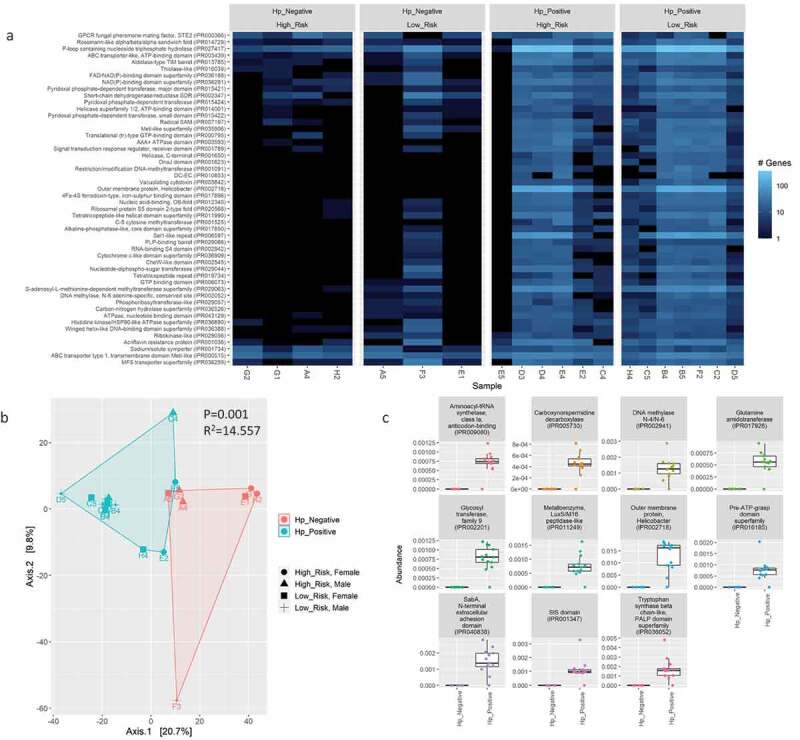
Comparison of InterProScan protein domain abundance in annotated bacterial genes by WMS after contig assembly with metaSpades using a heatmap of the 50 most abundant features (A) and PCA plot of Aitchison distance for beta diversity comparisons (B) between *H. pylori*-positive versus -negative samples. Statistical analysis for beta diversity was performed using PERMANOVA to determine significance differences (P-value) and percentage of the variance explained (R^2^) between the groups. C) Differential abundance analysis of InterProScan protein domains in annotated bacterial genes between *H. pylori* status determined using Welch’s t-test and generalized linear model (GLM) on centered log-ratio (clr) transformed abundances with a false discovery rate (FDR) correction for P-values using the Benjamini-Hochberg (BH) method. The abundance of differentially expressed features with a FDR≤0.01 for both tests is shown.

**Figure 9.**
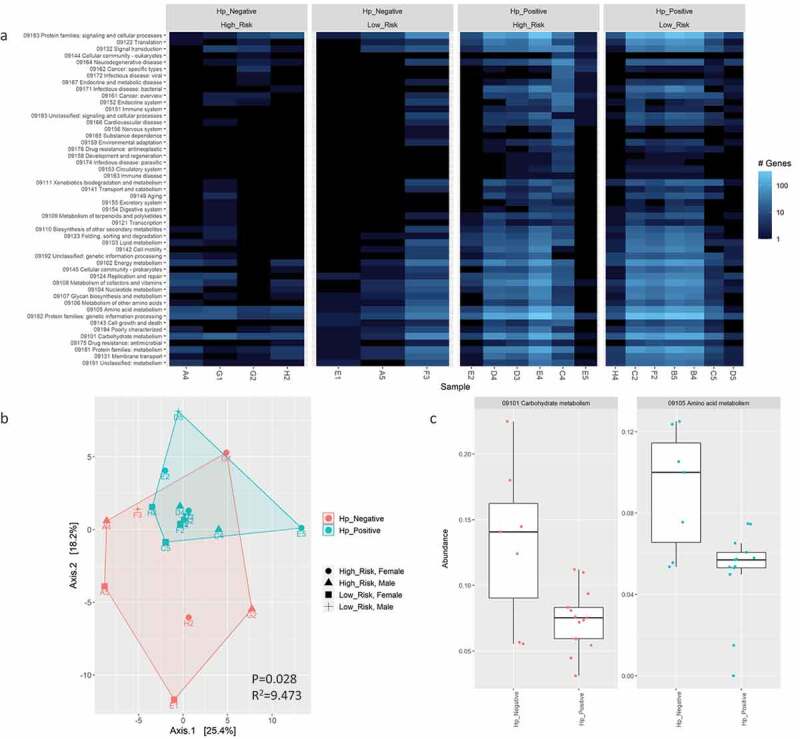
Comparison of KEGG metabolism pathway abundance in annotated bacterial genes by WMS after contig assembly with metaSpades using a heatmap of the 50 most abundant features (A) and PCA plot of Aitchison distance for beta diversity comparisons (B) between *H. pylori*-positive versus -negative samples. Statistical analysis for beta diversity was performed using PERMANOVA to determine significance differences (P-value) and percentage of the variance explained (R^2^) between the groups. C) Differential abundance analysis of KEGG pathways in annotated bacterial genes between *H. pylori* status determined using Welch’s t-test and generalized linear model (GLM) on centered log-ratio (clr) transformed abundances with a false discovery rate (FDR) correction for P-values using the Benjamini-Hochberg (BH) method. The abundance of differentially expressed features with a FDR≤0.05 for both tests is shown.

Gene annotations were analyzed using InterProScan, Gene Ontology (GO), and KEGG to evaluate and compare the functional potential of the *H. pylori*-positive versus *H. pylori*-negative gastric microbiomes. The beta diversity for these functional features was significantly different based on *H. pylori* status (Figures 8, 9, supplemental figure S15), but not risk groups or sexes (supplemental figures 16, 17, 18). Eleven InterProScan protein domains and 11 GO terms were significantly enriched in *H. pylori*-positive compared to *H. pylori*-negative samples based on differential abundance analysis by GLM and T-test (FDR<0.01; Figure 8C, supplemental figure S15C). The protein annotations corresponding to these enriched InterProScan protein domains and GO terms features were attributed to *H. pylori*. Two KEGG pathways (carbohydrate and amino acid metabolism) were statistically higher in *H. pylori*-negative samples (FDR<0.05; Figure 9C) and were attributed to over 15 different microbial taxa present at different abundances in each sample. The most commonly shared genera attributed to carbohydrate metabolism annotations in *H. pylori*-negative samples was *Neisseria* (*n* = 4/7; samples F3, A5, G2, and H2) followed by *Corynebacterium* (*n* = 2/7; samples E1 and G1). For amino acid metabolism genes, the most commonly shared genera in these samples was represented by *Bacillus* (*n* = 2/7; samples F3 and A5), *Corynebacterium* (*n* = 2/7; samples E1 and G1), *Propionibacterium*/*Cutibacterium* (*n* = 2/7; samples G1 and H2), and *Ralstonia* (*n* = 2/7; samples F3 and G2). No significant enrichments for InterProScan protein domains, GO terms, or KEGG pathways were detected between risk groups or sexes.

### Detection of *H.* *pylori* virulence factors gene sequences using WMS

**Figure 10.**
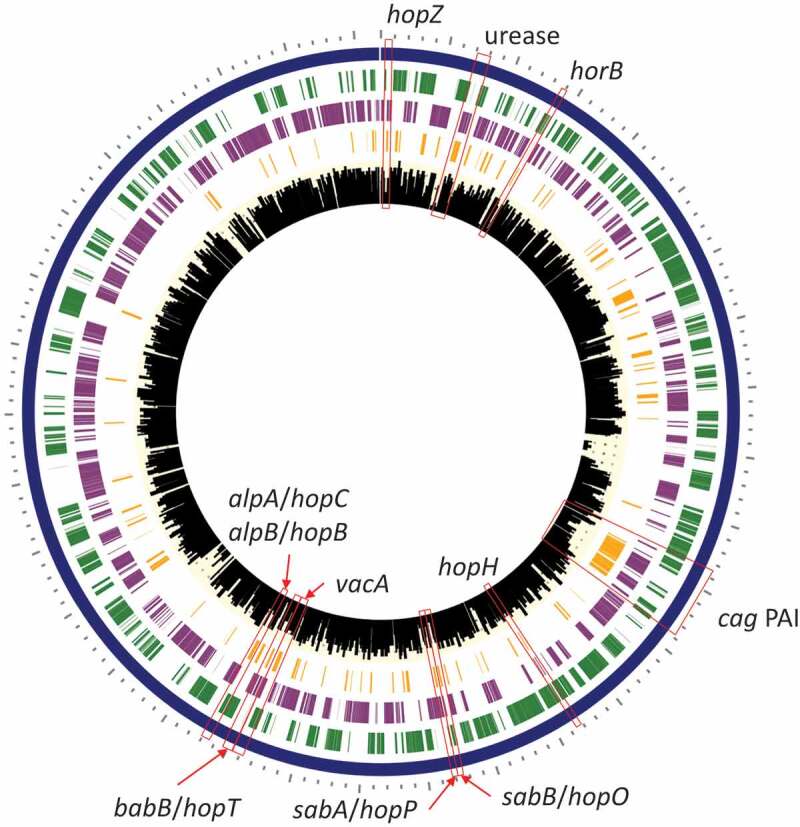
*H. pylori* virulence factor genes detected in assembled contigs and mapped to *H. pylori* reference strain 26695. Rings (outer to inner): 1) Forward protein coding genes (green), 2) Reverse protein coding genes (purple), 3) Virulence factor genes from *H. pylori* reference strain 26695 (orange), and 4) histogram showing number of genes detected by WMS per *H. pylori*-positive sample (*n* = 13).

Strain-specific *H. pylori* virulence factor genes detected by WMS were further evaluated. Across all 13 *H. pylori*-positive samples, more than 80% of the genes in the reference *H. pylori* strain 26695 genome were detected by WMS. Per sample, strain-specific virulence factor profiles were apparent, including differences in *cag* pathogenicity-associated island (PAI) and vacuolating cytotoxin (*vacA*) status as well as a repertoire of outer membrane protein including those belonging to Hop and Hor families. (Figure 10). While *vacA* sequences were detected in the majority of *H. pylori*-positive samples by WMS, the sequences were not long enough to identify subtypes.

Complete urease operons were detected in *H. pylori* protein annotations (Figure 10). Urease activity is essential for *H. pylori* to neutralize stomach acid and colonize the gut. Interestingly, other urease-like genes were not detected in non-*H. pylori* contigs or protein annotation (see urease activity GO: 0009039 in supplemental figure 15C). This suggests that these bacteria may utilize alternative mechanisms to neutralize stomach acid and enable colonization in the gastric environment. Alternatively, increased pH of the stomach acid as a result of gastric disease progression (i.e., NAG and MAG-IM) may have facilitated increased colonization of non-*H. pylori* species. Gastric pH was not measured in patients to evaluate this potential association.

## Discussion

Gastric cancer is the 5th most common cancer and the 4th most common cause of cancer death globally, especially in emerging countries like Colombia, South America where *H. pylori* infection can exceed 80%^20,21^. While *H. pylori* infection is the strongest risk factor associated with gastric disease, age, sex, smoking, diet, and hygiene may also modify risk^20^. Recently, non-*H. pylori* microbes present in the gastric microbiome have been appreciated as important factors that may modulate gastric disease states^1,2,22^. Previous studies have shown that non-*H. pylori* microbes can be readily cultured from the gastric tissue samples^14^. Furthermore, non-*H. pylori* microbes residing in the gastric niche have been appreciated to interfere with urease-breath test results^23^, implying sufficient colonization loads exist in the stomach to yield physiological effects on the host.

Eradication of *H. pylori* infection with antibiotics effectively reduces gastric disease and cancer risk. For example, INS-GAS mice, a transgenic mouse line that overexpresses human gastrin and has increased indices of gastric disease, have reduced gastric disease progression and severity when maintained under germfree conditions or when treated with antibiotics under conventional housing^24–26^. Additionally, antibiotic treatment that failed to completely clear *H. pylori* in human patients still significantly reduced their risk for gastric cancer^27^, implying that other microbes present in the gastric niche of the host may contribute to disease progression.

With the advent of next-generation sequencing technology, targeted 16S rRNA profiling has facilitated culture-free approaches to evaluate the gastric microbiome. Numerous studies have found that while *H. pylori* is the predominant bacterial taxa present in the stomach in infected individuals, there is a high diversity of non-*H. pylori* taxa also detectable^2,22^. In *H. pylori*-negative patients, the gastric microbiome remains highly diverse including a variety of bacteria taxa from all major phyla. Additionally, the magnitude of gastric microbiome diversity is also associated with the host states, such as increases in gastric pH due to acid suppressant drugs, antibiotic use, and during progression of disease noted in *H. pylori*-associated cancer^2,22,28–30^. While culture and 16S rRNA sequencing have provided insights into the diversity of bacterial microbes inhabiting the stomach, these approaches fail to capture the functional diversity of the gastric microbiome and how this may impact the host. In general, it is also largely unknown if non-bacterial taxa are present in the gastric niche and contribute to normal or pathological states in the host. To this end, we utilized non-targeted WMS to characterize the composition and function of the gastric microbiome in human patients from high- and low-risk gastric cancer populations residing in Colombia, South America. In addition to capturing the bacterial diversity and strain-specific genetic signatures that included annotation of microbial protein coding gene sequences, WMS allowed us to identify eukaryota, viral, and archaea microbes present in the gastric microbiome. We did not identify any statistical differences in eukaryota, viral, or archaea populations between *H. pylori* status, risk group, or sex. In our study, *Candida* spp. were detected in all gastric biopsy tissues samples by WMS, but were not isolated by culture, even though media we used for culture will support the growth of fungi. The lack of fungal growth possibly is due in part to the polymicrobial nature of the gastric biopsies where other bacteria colonizing this niche can inhibit slower growing fungi, as well as reducing fungal viability. Further, 16S-based bacterial analysis would not detect fungal presence, given 18S sequencing is used to identify fungi in tissue samples. Future studies investigating the role of fungi in gastric cancer progression are warranted. Interestingly, *Candida* spp. have been recently detected in colorectal cancer tumor DNA from human patients and associated with metastatic progression^31^.

Our current WMS analysis confirms previous culture-based and 16S rRNA microbiome studies that non-*H. pylori* bacteria are present in stomach tissues of Colombian patients from high- and low-gastric-cancer-risk cohorts^13^. While *H. pylori* is the predominant taxa in the stomach when present, significant differences exist the gastric microbial population between gastric cancer risk groups and sex, even when *H. pylori* is excluded from analyses (see supplemental figures S3 and S4). This finding reinforces the significance non-Helicobacter microbes may have in modulating gastric cancer risk and progression. Additional studies are needed to understand how the gastric microbiota contributes to cancer risk as well as interplays with other potential multifactorial attributes between Túquerres and Tumaco populations, for example, diet and sex, parasitic infections, and genetic characterization of *H. pylori*.

Several soil-associated bacterial taxa were abundant in the gastric microbiome. *Bacillus*, *Actinomyces*, and *Arthrobacter* spp. were detected by both WMS and culture in our study. Perturbations in presences of these taxa have been associated with gastric cancer risk^32–35^. Interestingly, the abundance of *Keratinibaculum* spp., thermophilic anaerobes originally isolated from soil^36^, was significantly higher in males versus females. Future studies will be needed to validate the presence and enrichment of *Keratinibaculum* spp. in the gastric niche as well as its association with sex and/or gastric disease progression, especially since males had significantly higher pathology scores compared to females. The significant differences in the gastric microbial beta diversity as well as enrichment of *Keratinibaculum* spp. between males versus females is particularly interesting considering males have a greater than 2-fold higher incidence and death rate of gastric cancer compared to females^20^. *Keratinibaculum* spp. have not been previously described in the gastric microbiome. It is plausible these soil-associated microbes were introduced from consumption of food or water sources. The role of these taxa in gastric carcinogenesis requires further study.

*Proteobacteria* and *Firmicutes* phyla predominated in the gastric microbiome. *Klebsiella* spp., belonging to the *Proteobacteria* phylum, were abundant in the gastric microbiome and increased levels have been previously associated with gastric cancer^37^. Several taxa from the *Firmicutes* phylum were enriched in the gastric microbiome and have been associated with gastric or intestinal cancer, including *Clostridium* ^38–41^, *Lactococcus* ^42^, *Staphylococcus* ^14^, and *Streptococcus* ^14^ spp.

*Staphylococcus* and *Streptococcus* species are considered commensals of the skin and oral sites. However, *Staphylococcus epidermidis* and *Streptococcus salivarius* isolated from stomach biopsy samples from the same Colombian cohort of the current study were capable of gastric colonization as well as modulated *H. pylori*-associated gastric pathology and host immune responses in a germfree INS-GAS mouse model of gastric cancer^14^. *Staphylococcus epidermidis* was cultured from both high- and low-risk gastric cancer patients, but *Staphylococcus epidermidis* was more often present in the stomach biopsies of low-risk gastric cancer patients^14^. Both strains exhibited urease activity and were able to maintain persistent colonization up to 5 months in the germfree mouse gastric niche alone or during co-infection with *H. pylori*. Neither strain affected *H. pylori* gastric colonization, but *Streptococcus salivarius* caused significantly higher gastric pathology than *H. pylori* only or *H. pylori* with *Staphylococcus epidermidis*. In a different study from our lab, we showed germfree INS-GAS colonized with a restricted intestinal microbiota that includes *Clostridium* spp. exacerbated gastric pathology and carcinogenesis due to *H. pylori* infection^43^. This study emphasizes how non-*H. pylori* stomach microflora play a role in the pathology of *H. pylori*-induced gastric cancer. In particular, this study reinforces previous investigations observing that oral commensal and opportunistic pathogenic microbes may have the potential to colonize the stomach and act as risk factors alongside *H. pylori* to influence chronic inflammation and carcinogenesis^44^. While future research is required to ascertain if taxa detected by next-generation sequencing approaches represent actively colonizing versus swallowed/transient microbes, we hypothesize some of these taxa have the potential to adhere to the mucosal surface and sustain active colonization in the gastric niche, as suggested by the previously published studies described above.

While only~0.03–0.2% of the total WMS reads were mapped to known bacterial sequences, we determined that cultured bacteria taxa from the gastric biopsies were more often detected by WMS than 16S rRNA analysis, suggesting that WMS may have stronger taxonomic resolution. Interestingly, *Atopobium* was the only genera detected by culture but not 16S rRNA or WMS profiling. We speculate that *Atopobium* strains may have low genetic abundance in the gastric biopsy samples that prevented detection by 16S rRNA or WMS profiling methods, but were enriched for growth by the culturing conditions/media. Sample preparation methods that deplete host DNA in human colonic biopsy samples before WMS have been shown to increase the bacterial-to-human DNA ratio without distorting microbiome profiles, thereby increasing sequencing coverage of the microbial genomes in tissues samples^19^. Application of similar bacterial DNA enrichment methods for gastric biopsy tissues may improve detection of underrepresented species as well as the taxonomic and functional analysis by WMS. Follow-up studies in larger cohorts of patients will be needed to validate that WMS provides superior accuracy and depth of microbiome analysis compared to traditional 16S rRNA approaches.

In our study, we found a poor correlation between the relative abundance at the genus level detected for 16S rRNA profiling versus WMS despite using DNA from the same extraction. This finding suggests these approaches may have different sensitivities or biases for taxonomic detection since 16S rRNA profiling relies on sequencing PCR amplicons while WMS targets bacterial genomic fragments. It has been well described in the literature that 16S rRNA profiling is susceptible to technical artifacts due to contamination as well as PCR amplification bias^17,18^. Conversely, WMS may be less prone to these caveats. Furthermore, the 16S rRNA gene is also susceptible to misidentification since some species (and genera like *Escherichia* and *Shigella*) can have nearly identical 16S rRNA sequences, but are considered different species based on the gold standard for microbial speciation, whole-genome sequence analysis (e.g., core gene phylogeny, average nucleotide identity, digital DNA-DNA hybridization)^45,46^. WMS may also identify genera differently since this method can use any genomic sequence to identify a taxon, instead of a single gene (i.e., 16S rRNA sequence).

A potential limitation of our 16S rRNA profiling is a negative extraction control or negative PCR control to monitor for potential background noise was not included. However, since 16S rRNA and WMS profiling used the same DNA extracts, overlapping contamination would have been expected using both sequencing approaches which is not supported by the poor correlation in OTU abundance between these methods. Our culture results suggest the predominant isolates cultured from these gastric biopsy samples were also detected by 16S rRNA and WMS. Additionally, the OTUs we have detected by both 16S rRNA and WMS have been described in previous studies characterizing the gastric microbiota from these Colombian cohorts as well as other populations^1,2,13^.

WMS enabled identification of statistically significant differences in functional and metabolic genes harbored by the gastric microbiome between *H. pylori*-positive versus -negative patients. Interestingly, carbohydrate and amino acid metabolism pathways were enriched in *H. pylori*-negative patients. Comparative genome analysis and metabolic experiments have shown *H. pylori* lacks the traditional glycolysis pathway used by bacteria to metabolize glucose into pyruvate and instead relies on alternative pathways to process carbohydrates^47^. Additionally, *H. pylori* has a limited gene content for *de novo* biosynthesis of the amino acids histidine, leucine, methionine, phenylalanine, and valine. As demonstrated experimentally, *H. pylori* instead requires media supplemented with these amino acids to grow in the absence of serum *in vitro*; presumably serum acts as transporters for these nutrients^47^. Enrichment of carbohydrate and amino acid metabolism genes in *H. pylori*-negative samples were mainly represented by *Bacillus*, *Corynebacterium*, *Cutibacterium*, *Neisseria*, and *Ralstonia* spp. The biological significance of enrichment in carbohydrate and amino acid metabolism genes in the gastric microbiota requires further investigation, especially how it may relate to the pathogenesis of gastric disease and cancers. Continued optimization of WMS methods for gastric tissues is needed to increase sequencing coverage for non-Helicobacter species occupying the gastric microbiota as well as further elucidate their genetic features.

As expected, *H. pylori* represented the predominant taxa in *H. pylori*-positive patients and significant enrichment for *H. pylori*-specific genes were detected according to InterProScan domains and GO terms. These included genes for DNA methylation for epigenetic modification^48^ (IPR002941, GO: 0008170, GO 00063006), outer membrane proteins^49^ (IPR002718, IPR040838), and urease activity (GO: 0009039). In particular, WMS was able to detect strain-specific *H. pylori* virulence factors genes that facilitate host colonization (urease, outer membrane proteins), immune evasion (outer membrane proteins), gastric epithelial injury (vacuolating cytotoxin, *cag* PAI), and promote oncogenesis (*cag* PAI)^50^.

Previously, our group performed whole genome sequencing on *H. pylori* isolates cultured from gastric biopsy tissues collected from the same high- and low-risk Colombian populations^15^, which included five samples also analyzed by WMS in the current study (samples B4, C2, C5, D3, and D4). These five *H. pylori* isolates were *cag*-positive and *vacA*-positive and harbored a variety of outer membrane proteins. WMS was able to detect *vacA* (3/5 samples), *cag* PAI (4/5 samples), and outer membrane proteins (4/5 samples) (data not shown). The gold standard for detecting and characterizing disease-associated *H. pylori* strains has been culture to isolate the organism from the stomach and enable subsequent biochemical, molecular and whole genome sequence characterization. However, given the fastidious nature of this species, this has been a challenging bottleneck. Additionally, culture and passage of *H. pylori* may promote genotypic drifts given the high genomic plasticity of this species, which may confound associations between *H. pylori* genetics and host disease progression^51^. Therefore, WMS may augment traditional culture and genome sequencing approaches to identify, monitor, and characterize risk factors attributed to *H. pylori* colonization in patients, including detection of numerous strain-specific genes including disease-promoting virulence factors like *vacA* and *cag* PAI^52^. While contig sizes and protein gene annotations from non-*H. pylori* taxa were too low to appreciably characterize their strain-specific features and describe the microbiome beyond the genus level, continued optimization of WMS methods for gastric tissues, such as depletion of host DNA, may improve microbial genome sequencing coverage and therefore contig assembly for *H. pylori* and other taxa.

While numerous 16S rRNA profiling studies have been published, to our knowledge the gastric microbiome has been characterized using WMS in only a single report in the literature by Hu et al.^53^ In this study, the authors evaluated gastric wash samples from 6 patients with advanced gastric adenocarcinoma and 5 patients with superficial gastritis and found significant differences in the composition and functional pathways related to bacterial OTUs. The authors did not report characterization of non-bacterial taxa. Unlike the study by Hu et al., we surveyed the gastric microbiome using gastric biopsy samples, which represented mucosal-associated microbes. Host-microbe interactions at the mucosal surface in the stomach and other sites of the gastrointestinal tract are hypothesized to be more influential for host homeostasis and disease progression since microbes are in closer proximity to the epithelial barrier and immune sites compared to organisms primarily residing in the luminal content^54^. In the study by Hu et al., the authors did not perform 16S rRNA or culture profiling of the gastric microbiota for comparison with WMS.

In a different published report by Thorell et al., metatranscriptomic RNA sequencing of stomach biopsy tissues from Nicaragua patients with different *H. pylori* infection statuses and premalignant tissue changes was performed to evaluate the composition of the transcriptionally active microbial community in the gastric microbiota^55^. Similar to our findings, these authors determined that RNA transcripts from *H. pylori* predominated in *H. pylori*-positive samples, while fungi, *Bacteroidetes, Firmicutes*, and *Actinobacteria* transcripts were also detected in biopsy tissues as well. Additionally, the authors noted highly expressed *H. pylori* nickel transport genes which parallels our findings of genes enriched for nickel cation binding (GO:0016151) in *H. pylori*-positive samples. Significant differences in stomach microbiota at different stages of gastric disease progression were not noted in this study. While these authors performed 16S rRNA profiling to support taxa identified by metatranscriptomic RNA sequencing, the authors did not statistically analyze the agreement of taxa identified between sequencing methods.

In conclusion, our study demonstrates that WMS is a feasible approach to characterize the gastric microbiome. By continuing to study the gastric microbiome with WMS, novel insights into the structure, function, and interactions of *H. pylori* with other microorganisms and host disease risk may be elucidated. Future studies comparing WMS versus culture and 16S rRNA profiling in larger sample populations are warranted to optimize WMS as a novel approach to detect and characterize non-*H. pylori* taxa present in the stomach. By integrating next-generation sequencing approaches with traditional culture techniques, future studies will have the potential to identify specific microbes associated with augmented or suppressed risk for stomach disease in human patients.

## Methods

### Study population, samples, and histopathology

As previously cited, subjects between 40 and 60 years of age with dyspeptic symptoms that warranted upper gastrointestinal tract endoscopy were recruited in Tumaco (LGCR) and Túquerres (HGCR) in 2010^14,15^. Subjects that had received proton pump inhibitors, H2-receptor antagonists, or antimicrobials during the 30-day period previous to the endoscopic procedure were excluded from this study. Other exclusion criteria were major diseases or previous gastrectomy. Participation was voluntary and informed consent was obtained from all participants. The Ethics Committees of the participating hospitals in Nariño and the Universidad del Valle in Cali, Colombia, and the Institutional Review Board of Vanderbilt University approved all study protocols, and all experiments were performed in accordance with the relevant guidelines and regulations. A total of 163 participants were recruited (81 from the HGCR and 82 from the LGCR) and a sample population of 20 patients (10 from HGCR, 10 from LGCR) consisting of 10 males and 10 females was selected for this study. Four biopsy samples (2 antrum, 1 incisura, and 1 corpus) were used for histopathology, one frozen antral biopsy was used for *H. pylori* culture, and another frozen antral biopsy for both DNA extraction and culture. By histologic diagnosis, 17 patients had non-atrophic gastritis (NAG) and 3 had multifocal atrophic gastritis with intestinal metaplasia (MAG-IM) (Table 1, supplemental table S19). Thirteen of the patients were *H. pylori*-positive by a modified Steiner stain and antral culture (Table 1).

### 16S rRNA sequencing and WMS

DNA was extracted from antral gastric biopsy samples using the DNeasy PowerLyzer PowerSoil Kit (Qiagen, Germantown, MD) following the manufacture’s protocol. DNA was stored at −20°C until use. 16S rRNA library preparations and sequencing were performed as previously described^56^. WMS libraries were prepared from>5 ug of DNA per sample using NexteraFlex and sequenced by 2 × 250 bp paired reads on an Illumina Novaseq SP500.

### Bioinformatic analysis

All scripts for the bioinformatic workflow were performed using default parameters unless stated otherwise and have also been deposited in GitHub (https://github.com/TonyMannion/WMS_Gastric_Biopsies). 16S rRNA and WMS reads were decontaminated for low-quality base pairs and adapter sequence using BBDuk version 38.90 (sourceforge.net/projects/bbmap/) before taxonomic classification. Decontaminated 16S rRNA reads were analyzed using Kraken2^57^ against the SILVA 138 database, a highly curated and comprehensive database of 16S rRNA gene sequences (available at https://benlangmead.github.io/aws-indexes/k2). Decontaminated WMS reads analyzed by Kraken2^58^ followed by Bracken^59^ for taxonomic classification and abundance against more than 34,000 RefSeq genomes from archaea, bacteria, fungi, protozoa, eukaryota, human, and viral species (PlusPF: standard plus protozoa & fungi and EuPathDB46: eukaryotic pathogen genomes with contaminants removed; available at https://benlangmead.github.io/aws-indexes/k2). This database includes more than 60 different strains of *H. pylori*. Bacterial reads were extracted using Kraken2 from decontaminated WMS reads and assembled into contigs using metaSPAdes^60^. BBMap version 38.90 (sourceforge.net/projects/bbmap/) was used to estimate contig coverage by mapping bacterial reads to assembled contig sequences, and no contigs were excluded based on coverage prior to gene annotation with RAST hosted by PATRIC^61^. DIAMOND^62^ analysis against the nr database was used to predict taxonomy for protein gene annotations. InterProScan version 5.52–86.0^63^ and KAAS^64^ (program: GHOSTX; method: BBG; GENES data set: hsa, dme, ath, sce, pfa, eco, sty, hin, pae, nme, hpy, rpr, mlo, bsu, sau, lla, spn, cac, mge, mtu, ctr, bbu, syn, aae, mja, afu, pho, ape) were used to analyze gene annotations for protein domains and KEGG pathways, respectively. GO terms were included in the outputs from InterProScan domain analysis. Protein gene annotations with ≥90% percent identity to the *Helicobacter pylori* 26695 genome (Genbank: AE000511) based DIAMOND analysis were mapped and visualized on the circular chromosome using PATRIC.

### Bacterial culture methods for gastric biopsies from patients

Antral biopsies were frozen at −80°C in thioglycolate containing 20% glycerol. The biopsies were thawed in an anaerobic atmosphere (10% CO_2_, 10% H_2_, 80% N_2_), and were homogenized in Brain Heart Infusion (BHI) with 20% glycerol with tissue grinders. The homogenate was divided into aliquots to isolate bacteria under diverse culture conditions. For aerobic culture, the homogenates were plated onto chocolate agar, blood agar, MacConkey agar, and Brucella broth medium containing 10% FCS. The plates were incubated at 37°C in 5% CO_2_ for 24–48 hours. For anaerobic culture, the homogenates were plated onto pre-reduced Brucella blood agar plates (BBL) and inoculated into thioglycolate broth. The cultures were incubated at 37°C in an anaerobic chamber (Coy Lab Products) with mixed gas (10% CO_2_, 10% H_2_, 80% N_2_) for 48 hours. For microaerobic culture to detect the growth of *H. pylori*, the homogenates were plated onto *H. pylori*-selective plates and Brucella blood agar plates after passing through 0.65 µm syringe filter. The plates were placed into a vented jar filled with mixed gas (10% CO_2_, 10% H_2_, 80% N_2_) and incubated at 37°C for up to 3 weeks. The plates were checked every 2–3 days for growth. All bacterial strains isolated from the different culture conditions were identified by 16S rRNA sequencing.

### Statistical analysis

The Phyloseq, Vegan, and Microbiome packages in R were used to plot and statistically analyze 16S rRNA and shotgun metagenomic data, as described previously^56^. Briefly, alpha diversity was analyzed by the Observed and Chao1 metrics to describe the species richness (i.e., “how many microbes in a sample?”) and the Shannon and Simpson metrics to describe species evenness (i.e., “how are microbes balanced to each other in a sample?”). For beta diversity analyses, the Aitchison distance (i.e., the Euclian distance between samples after centered log-ratio (clr) transformation of abundances)^65^ was plotted on PCA plots and permutational ANOVA (PERMANOVA) using the adonis function in Vegan. To identify differentially abundant features, the Welch’s t-test and generalized linear model (GLM) on clr transformed abundances with a false discovery rate (FDR) correction for P-values using the Benjamini-Hochberg (BH) method. A P-value or FDR≤0.05 was considered statistically significant. All scripts for these analyses were performed using default parameters unless stated otherwise and have also been deposited in GitHub (https://github.com/TonyMannion/WMS_Gastric_Biopsies).

## Supplementary Material

Supplemental Material

## Data Availability

The authors confirm that the data supporting the findings of this study are available within the article and the raw sequencing results can be accessed with the accession number PRJNA861967. https://www.ncbi.nlm.nih.gov/bioproject/PRJNA861967
