# Supplementary material for: Shotgun Metagenomics of Gastric Biopsies Reveals Compositional and Functional Microbiome Shifts in High- and Low-Gastric-Cancer-Risk Populations from Colombia, South America
Source: Gut Microbes. 2023 Mar 12;15(1):2186677. doi: 10.1080/19490976.2023.2186677 (PMC10026914; doi:10.1080/19490976.2023.2186677)
Supplement: Supplemental Material [file KGMI_A_2186677_SM5315.pptx]

## Slide 1
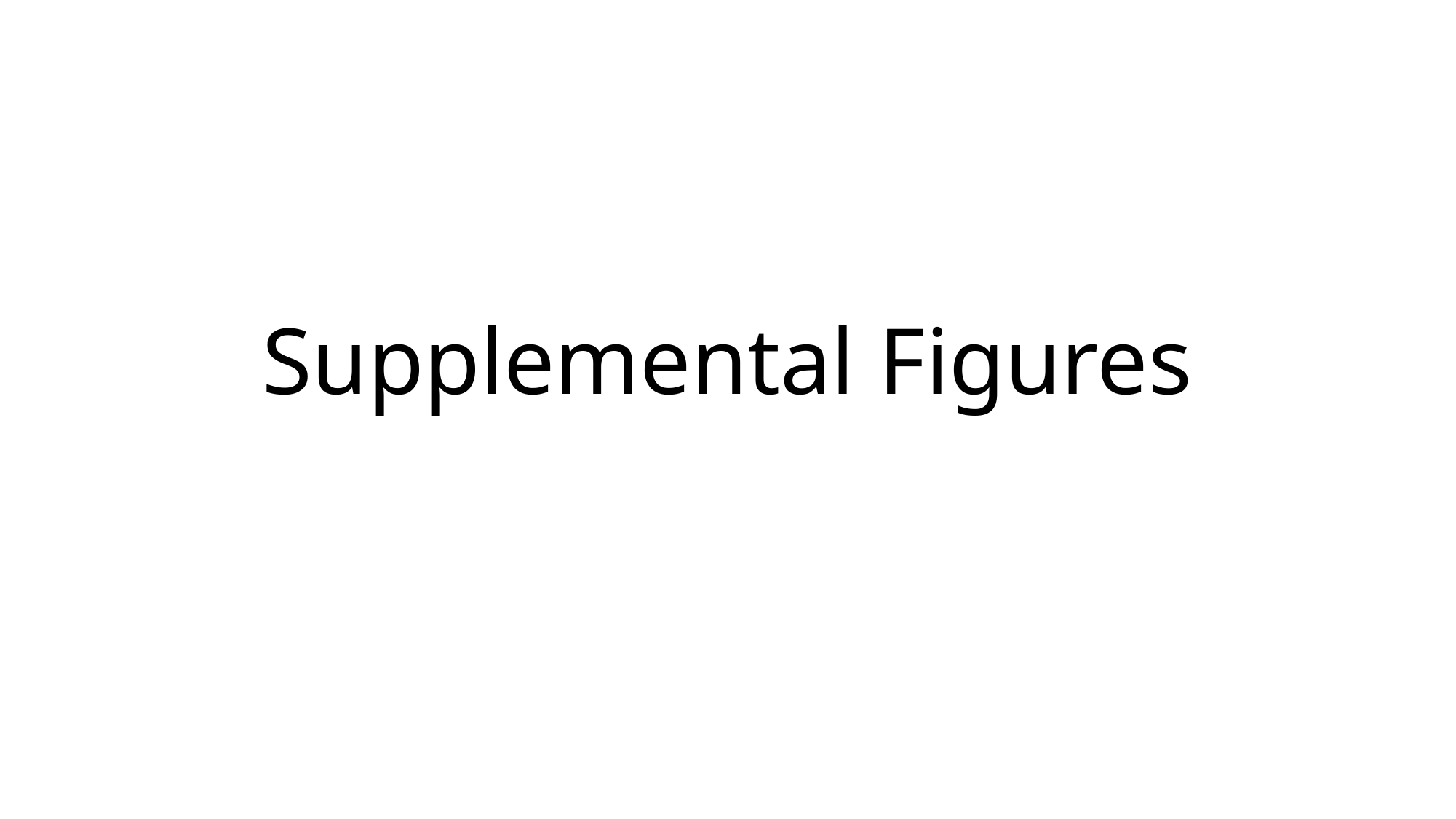

# Supplemental Figures

## Slide 2
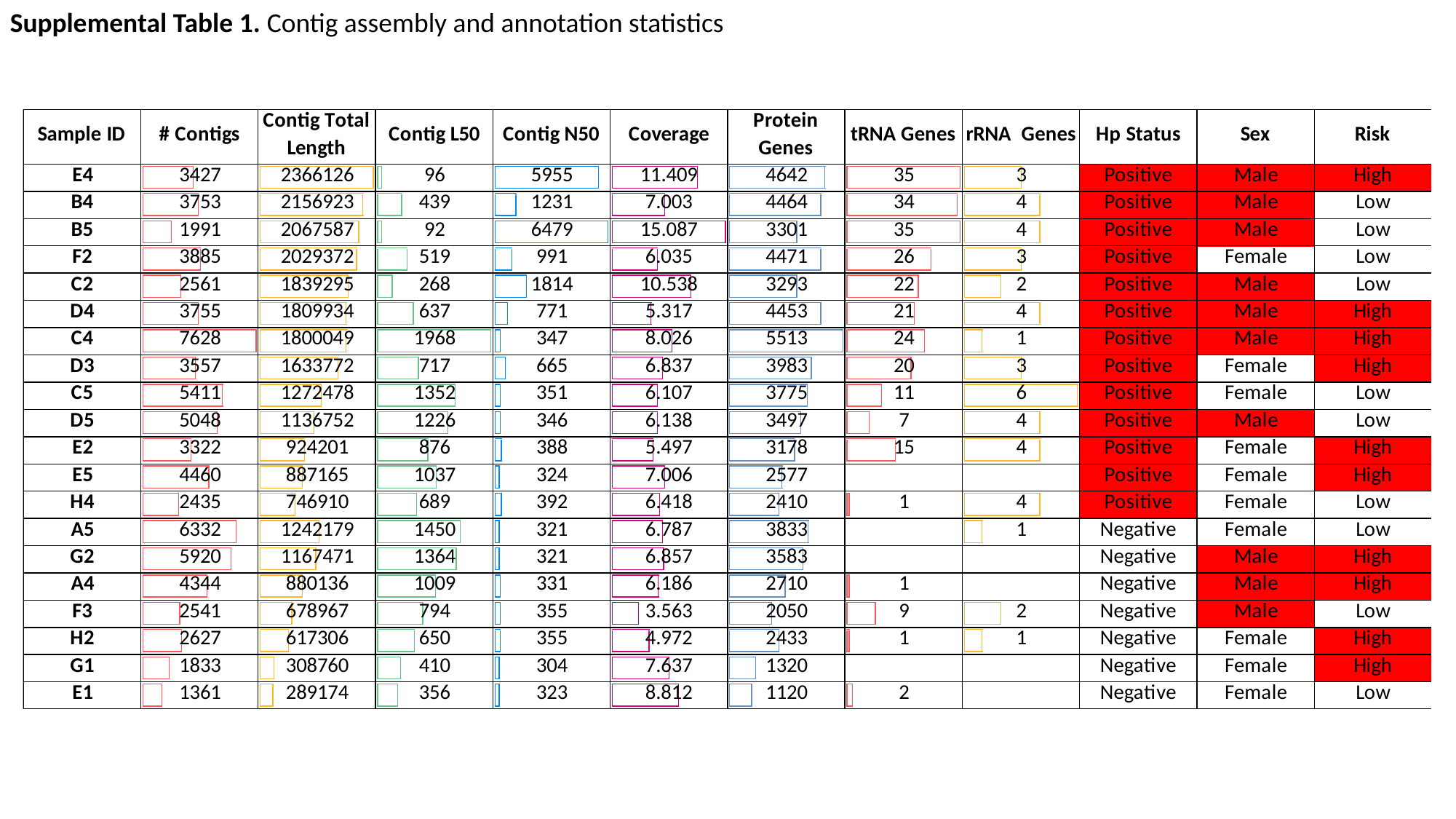

Supplemental Table 1. Contig assembly and annotation statistics

## Slide 3
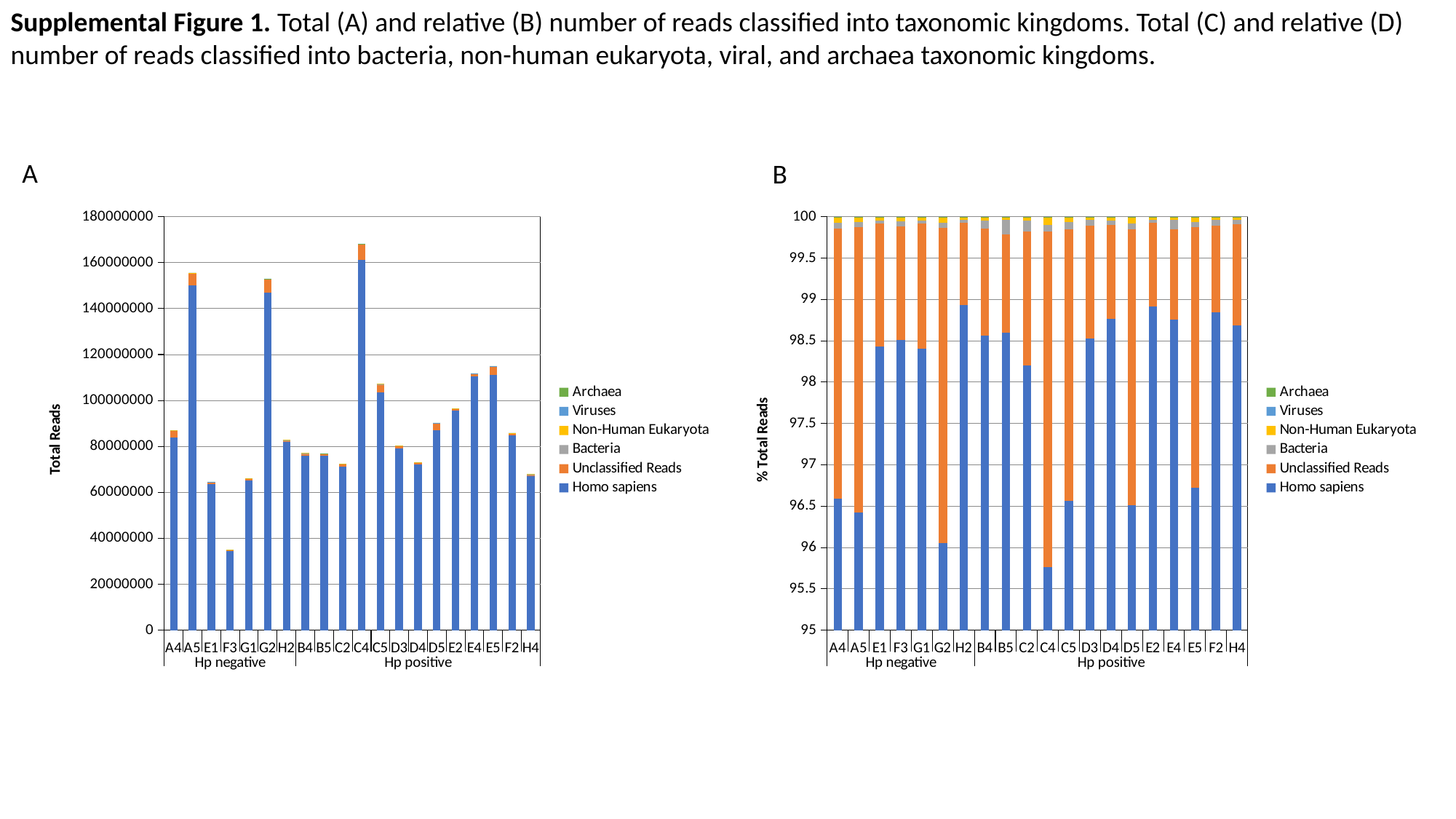

Supplemental Figure 1. Total (A) and relative (B) number of reads classified into taxonomic kingdoms. Total (C) and relative (D) number of reads classified into bacteria, non-human eukaryota, viral, and archaea taxonomic kingdoms.
A
B
### Chart
| Category | Homo sapiens | Unclassified Reads | Bacteria | Non-Human Eukaryota | Viruses | Archaea |
|---|---|---|---|---|---|---|
| A4 | 84038395.0 | 2836376.0 | 61394.0 | 62492.0 | 2363.0 | 1603.0 |
| A5 | 150105685.0 | 5364562.0 | 95117.0 | 96172.0 | 3746.0 | 2481.0 |
| E1 | 63569912.0 | 963590.0 | 24921.0 | 25760.0 | 1353.0 | 565.0 |
| F3 | 34539378.0 | 480353.0 | 23021.0 | 17912.0 | 961.0 | 425.0 |
| G1 | 65043082.0 | 1002099.0 | 25468.0 | 26130.0 | 1611.0 | 540.0 |
| G2 | 146978284.0 | 5824790.0 | 102383.0 | 101058.0 | 3863.0 | 2792.0 |
| H2 | 81884896.0 | 828344.0 | 26729.0 | 29120.0 | 2052.0 | 540.0 |
| B4 | 75980290.0 | 1000353.0 | 73693.0 | 33802.0 | 2183.0 | 630.0 |
| B5 | 75951121.0 | 913300.0 | 139467.0 | 24639.0 | 1856.0 | 618.0 |
| C2 | 71122649.0 | 1173773.0 | 96457.0 | 30258.0 | 1728.0 | 653.0 |
| C4 | 161108238.0 | 6818394.0 | 131990.0 | 163823.0 | 5092.0 | 3393.0 |
| C5 | 103527838.0 | 3515637.0 | 92279.0 | 63627.0 | 6712.0 | 1698.0 |
| D3 | 79172667.0 | 1093016.0 | 56281.0 | 31382.0 | 1739.0 | 646.0 |
| D4 | 72143872.0 | 825181.0 | 43534.0 | 29120.0 | 1903.0 | 572.0 |
| D5 | 87077933.0 | 3012230.0 | 66260.0 | 67647.0 | 2391.0 | 1623.0 |
| E2 | 95476797.0 | 975877.0 | 33310.0 | 32888.0 | 2076.0 | 657.0 |
| E4 | 110433124.0 | 1222199.0 | 122251.0 | 43090.0 | 3034.0 | 911.0 |
| E5 | 111291985.0 | 3628692.0 | 69311.0 | 67036.0 | 2601.0 | 1765.0 |
| F2 | 84813397.0 | 898438.0 | 55602.0 | 30902.0 | 2991.0 | 673.0 |
| H4 | 66995452.0 | 828060.0 | 33889.0 | 24532.0 | 2117.0 | 503.0 |
### Chart
| Category | Homo sapiens | Unclassified Reads | Bacteria | Non-Human Eukaryota | Viruses | Archaea |
|---|---|---|---|---|---|---|
| A4 | 96.59294410008765 | 3.260104008588339 | 0.07056568857699842 | 0.07182771949300885 | 0.002716010067880367 | 0.001842473186124515 |
| A5 | 96.42695578531568 | 3.446161168256783 | 0.0611025675238874 | 0.0617802929435043 | 0.002406407034962017 | 0.0015937789251844006 |
| E1 | 98.42661349072613 | 1.4919463864214377 | 0.038585701279598844 | 0.03988474238443346 | 0.002094877967629599 | 0.0008748012207765879 |
| F3 | 98.50929423693138 | 1.370008313832192 | 0.06565788366624313 | 0.05108657366012541 | 0.00274085514109985 | 0.0012121367689567496 |
| G1 | 98.40262467183659 | 1.5160593371178626 | 0.03853012446646262 | 0.03953165353811325 | 0.002437255792189072 | 0.0008169572487784597 |
| G2 | 96.05596956131292 | 3.8067246106985433 | 0.06691123385000128 | 0.06604529531673646 | 0.0025246192860392345 | 0.0018246795357549942 |
| H2 | 98.92863719899563 | 1.0007577350035939 | 0.03229244552856188 | 0.03518111466166792 | 0.00247910876668072 | 0.0006523970438633474 |
| B4 | 98.55928486340764 | 1.297627006832488 | 0.0955922829386292 | 0.043846910125677395 | 0.0028317201586993005 | 0.0008172165368669534 |
| B5 | 98.59812285186324 | 1.1856265505364523 | 0.1810530801748247 | 0.03198582347385048 | 0.0024094195530446243 | 0.0008022743985892122 |
| C2 | 98.20109122312387 | 1.6206622091401544 | 0.13318095978271083 | 0.0417780926330413 | 0.00238589940081616 | 0.0009016159194056437 |
| C4 | 95.76612219881326 | 4.05299667546271 | 0.07845762964039965 | 0.09737983377967416 | 0.0030267918033859767 | 0.0020168705005672856 |
| C5 | 96.56745749009976 | 3.2792737983007227 | 0.08607490103028054 | 0.05934923143785325 | 0.006260739016626132 | 0.0015838401147543466 |
| D3 | 98.52771671008755 | 1.3602215876799129 | 0.07003980836164629 | 0.039053841722875994 | 0.0021641269121178176 | 0.0008039252358988558 |
| D4 | 98.76744461317945 | 1.129701199200232 | 0.0595995448343853 | 0.03986628257401801 | 0.0026052725184875095 | 0.000783087693418211 |
| D5 | 96.50868015772119 | 3.3384616700937593 | 0.073436115522524 | 0.07497333091989408 | 0.00264995098421906 | 0.0017987747584222225 |
| E2 | 98.9175397570316 | 1.011045143727148 | 0.03451040831739174 | 0.03407320050262322 | 0.0021508137996669244 | 0.0006806766215708907 |
| E4 | 98.75565404391443 | 1.0929606737994497 | 0.10932387878950688 | 0.03853355749269823 | 0.002713177382985529 | 0.0008146686209294056 |
| E5 | 96.72400533315303 | 3.1537008200578835 | 0.060238278018369146 | 0.058261072632618115 | 0.002260532399269642 | 0.0015339637388354164 |
| F2 | 98.84780545274683 | 1.0471060914510353 | 0.06480268298631676 | 0.03601547623544406 | 0.003485932606957905 | 0.0007843639734144667 |
| H4 | 98.69027494369743 | 1.219806220127869 | 0.049921518964704675 | 0.03613782357821521 | 0.003118529778048329 | 0.0007409638537356208 |

## Slide 4
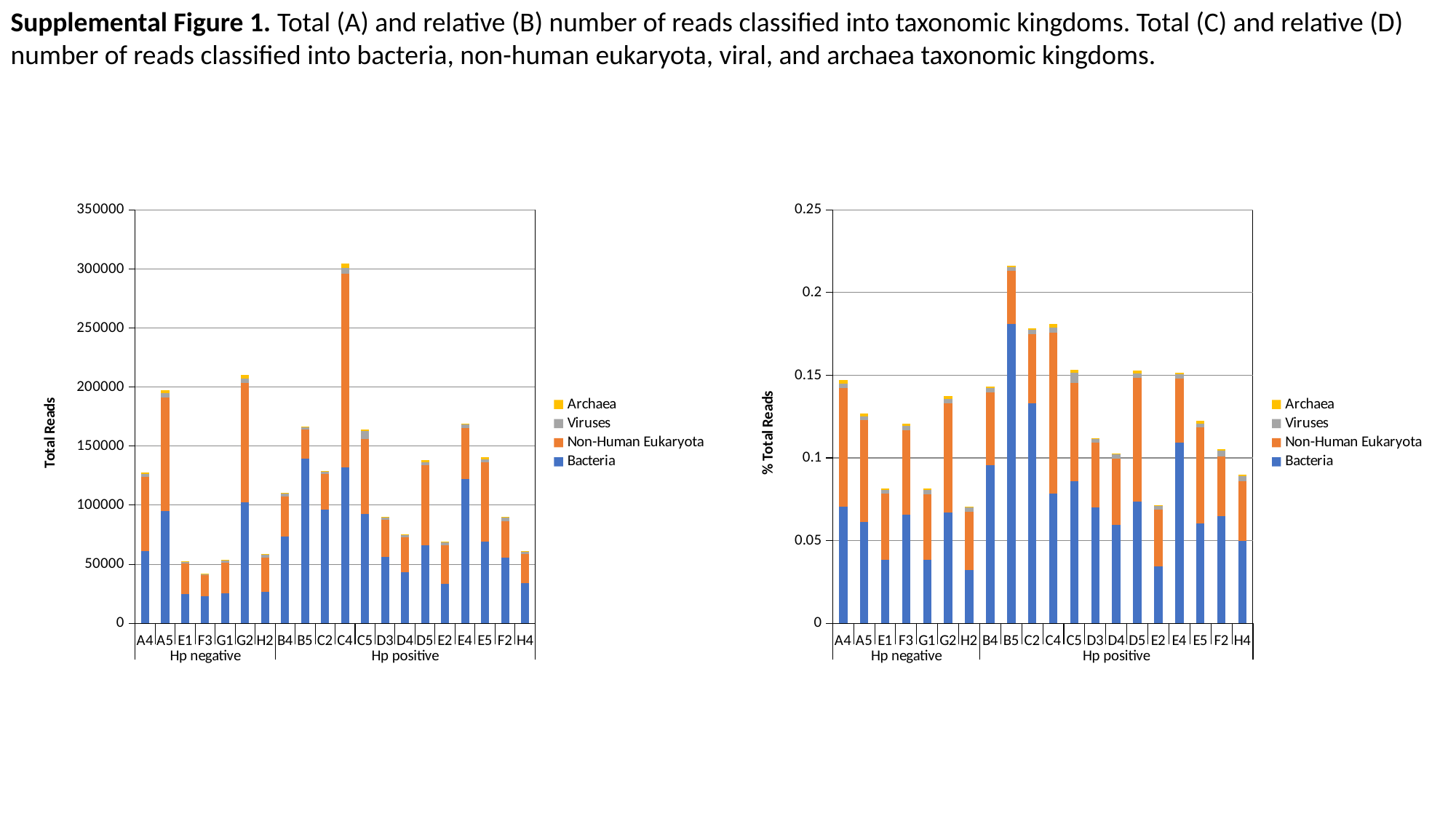

Supplemental Figure 1. Total (A) and relative (B) number of reads classified into taxonomic kingdoms. Total (C) and relative (D) number of reads classified into bacteria, non-human eukaryota, viral, and archaea taxonomic kingdoms.
### Chart
| Category | Bacteria | Non-Human Eukaryota | Viruses | Archaea |
|---|---|---|---|---|
| A4 | 61394.0 | 62492.0 | 2363.0 | 1603.0 |
| A5 | 95117.0 | 96172.0 | 3746.0 | 2481.0 |
| E1 | 24921.0 | 25760.0 | 1353.0 | 565.0 |
| F3 | 23021.0 | 17912.0 | 961.0 | 425.0 |
| G1 | 25468.0 | 26130.0 | 1611.0 | 540.0 |
| G2 | 102383.0 | 101058.0 | 3863.0 | 2792.0 |
| H2 | 26729.0 | 29120.0 | 2052.0 | 540.0 |
| B4 | 73693.0 | 33802.0 | 2183.0 | 630.0 |
| B5 | 139467.0 | 24639.0 | 1856.0 | 618.0 |
| C2 | 96457.0 | 30258.0 | 1728.0 | 653.0 |
| C4 | 131990.0 | 163823.0 | 5092.0 | 3393.0 |
| C5 | 92279.0 | 63627.0 | 6712.0 | 1698.0 |
| D3 | 56281.0 | 31382.0 | 1739.0 | 646.0 |
| D4 | 43534.0 | 29120.0 | 1903.0 | 572.0 |
| D5 | 66260.0 | 67647.0 | 2391.0 | 1623.0 |
| E2 | 33310.0 | 32888.0 | 2076.0 | 657.0 |
| E4 | 122251.0 | 43090.0 | 3034.0 | 911.0 |
| E5 | 69311.0 | 67036.0 | 2601.0 | 1765.0 |
| F2 | 55602.0 | 30902.0 | 2991.0 | 673.0 |
| H4 | 33889.0 | 24532.0 | 2117.0 | 503.0 |
### Chart
| Category | Bacteria | Non-Human Eukaryota | Viruses | Archaea |
|---|---|---|---|---|
| A4 | 0.07056568857699842 | 0.07182771949300885 | 0.002716010067880367 | 0.001842473186124515 |
| A5 | 0.0611025675238874 | 0.0617802929435043 | 0.002406407034962017 | 0.0015937789251844006 |
| E1 | 0.038585701279598844 | 0.03988474238443346 | 0.002094877967629599 | 0.0008748012207765879 |
| F3 | 0.06565788366624313 | 0.05108657366012541 | 0.00274085514109985 | 0.0012121367689567496 |
| G1 | 0.03853012446646262 | 0.03953165353811325 | 0.002437255792189072 | 0.0008169572487784597 |
| G2 | 0.06691123385000128 | 0.06604529531673646 | 0.0025246192860392345 | 0.0018246795357549942 |
| H2 | 0.03229244552856188 | 0.03518111466166792 | 0.00247910876668072 | 0.0006523970438633474 |
| B4 | 0.0955922829386292 | 0.043846910125677395 | 0.0028317201586993005 | 0.0008172165368669534 |
| B5 | 0.1810530801748247 | 0.03198582347385048 | 0.0024094195530446243 | 0.0008022743985892122 |
| C2 | 0.13318095978271083 | 0.0417780926330413 | 0.00238589940081616 | 0.0009016159194056437 |
| C4 | 0.07845762964039965 | 0.09737983377967416 | 0.0030267918033859767 | 0.0020168705005672856 |
| C5 | 0.08607490103028054 | 0.05934923143785325 | 0.006260739016626132 | 0.0015838401147543466 |
| D3 | 0.07003980836164629 | 0.039053841722875994 | 0.0021641269121178176 | 0.0008039252358988558 |
| D4 | 0.0595995448343853 | 0.03986628257401801 | 0.0026052725184875095 | 0.000783087693418211 |
| D5 | 0.073436115522524 | 0.07497333091989408 | 0.00264995098421906 | 0.0017987747584222225 |
| E2 | 0.03451040831739174 | 0.03407320050262322 | 0.0021508137996669244 | 0.0006806766215708907 |
| E4 | 0.10932387878950688 | 0.03853355749269823 | 0.002713177382985529 | 0.0008146686209294056 |
| E5 | 0.060238278018369146 | 0.058261072632618115 | 0.002260532399269642 | 0.0015339637388354164 |
| F2 | 0.06480268298631676 | 0.03601547623544406 | 0.003485932606957905 | 0.0007843639734144667 |
| H4 | 0.049921518964704675 | 0.03613782357821521 | 0.003118529778048329 | 0.0007409638537356208 |

## Slide 5
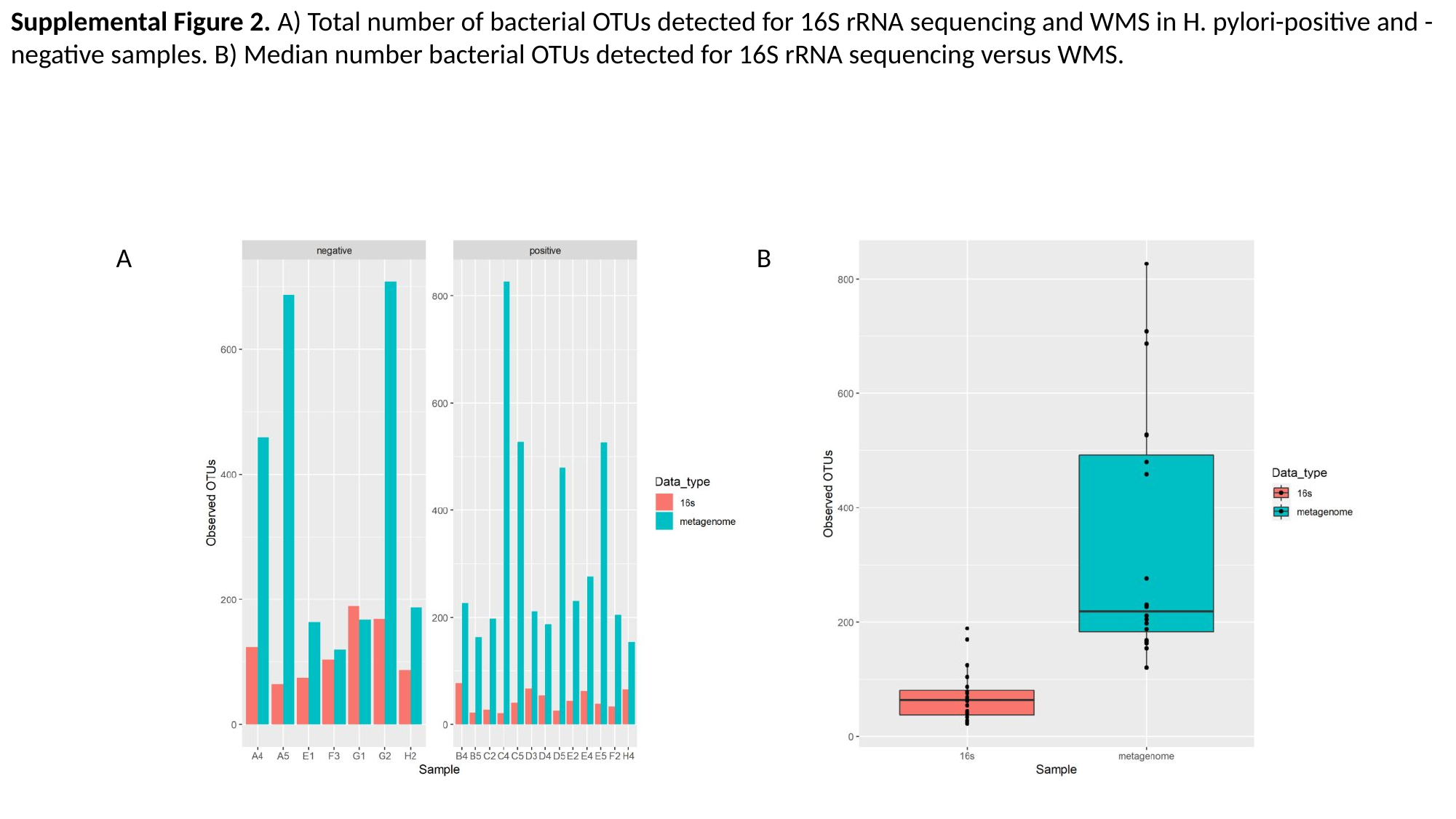

Supplemental Figure 2. A) Total number of bacterial OTUs detected for 16S rRNA sequencing and WMS in H. pylori-positive and -negative samples. B) Median number bacterial OTUs detected for 16S rRNA sequencing versus WMS.
A
B

## Slide 6
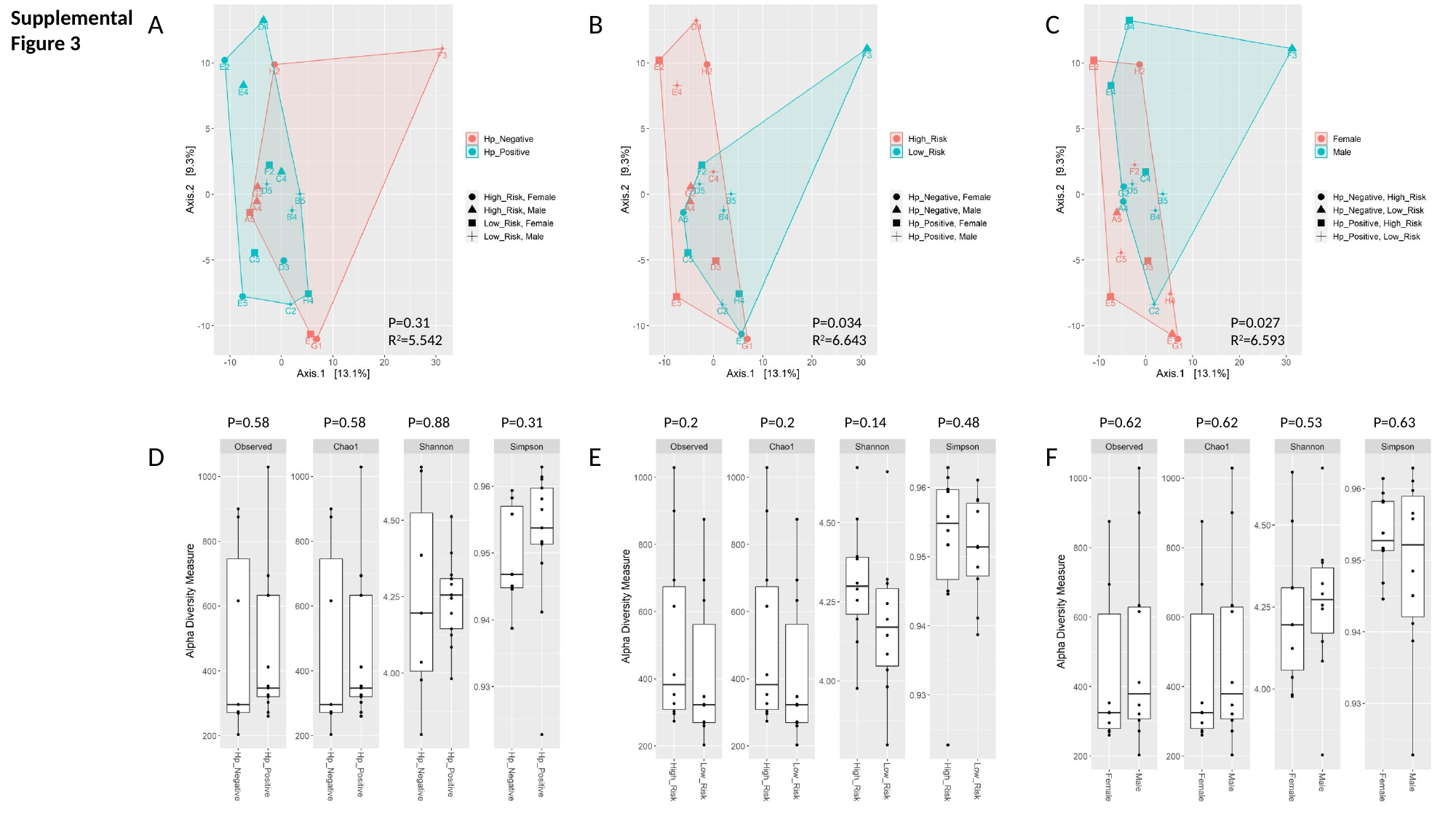

Supplemental Figure 3
A
B
C
P=0.31
R2=5.542
P=0.034
R2=6.643
P=0.027
R2=6.593
P=0.58
P=0.58
P=0.88
P=0.31
P=0.2
P=0.2
P=0.14
P=0.48
P=0.62
P=0.62
P=0.53
P=0.63
D
E
F

## Slide 7
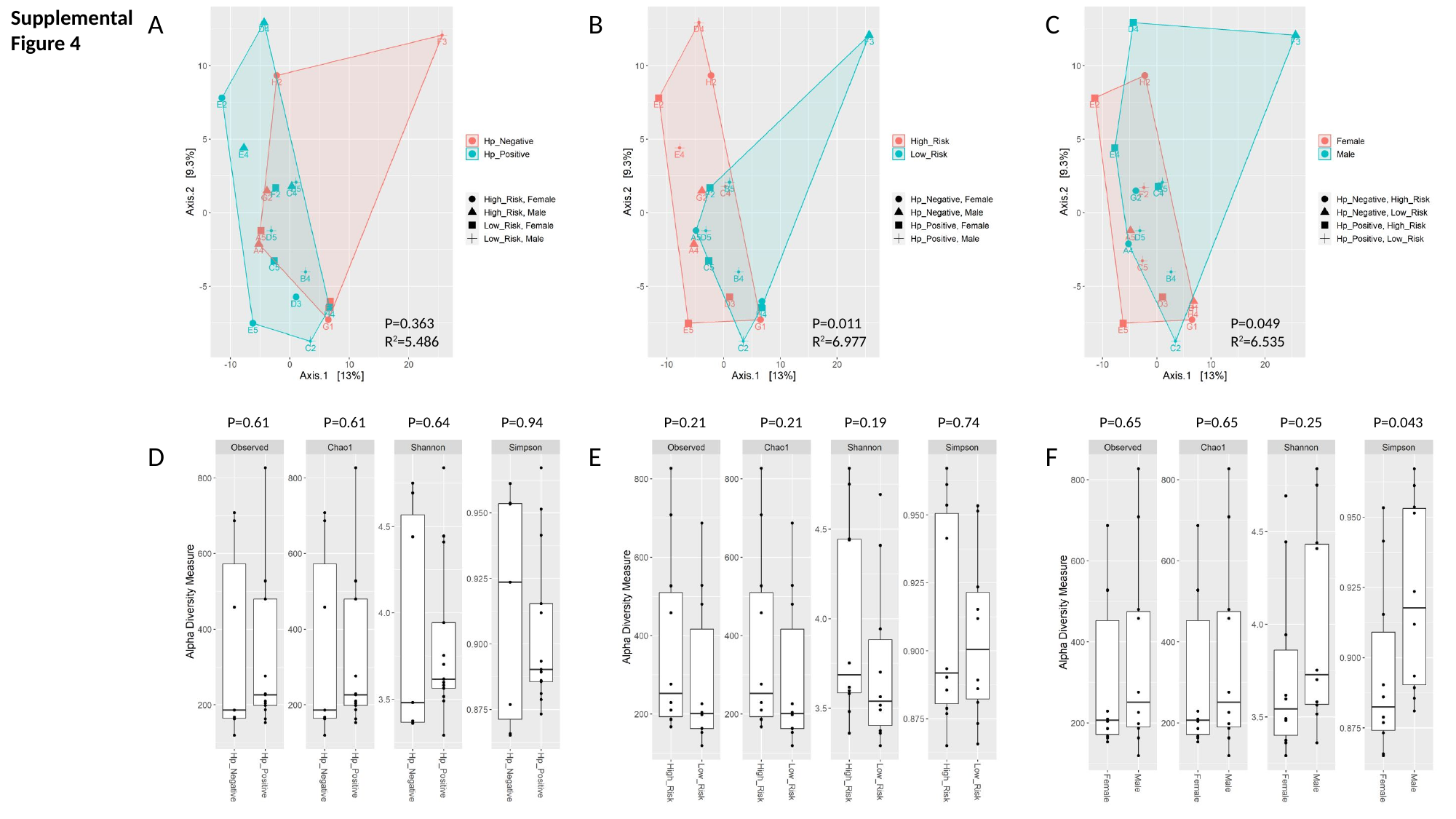

Supplemental Figure 4
A
B
C
P=0.363
R2=5.486
P=0.011
R2=6.977
P=0.049
R2=6.535
P=0.61
P=0.61
P=0.64
P=0.94
P=0.21
P=0.21
P=0.19
P=0.74
P=0.65
P=0.65
P=0.25
P=0.043
D
E
F

## Slide 8
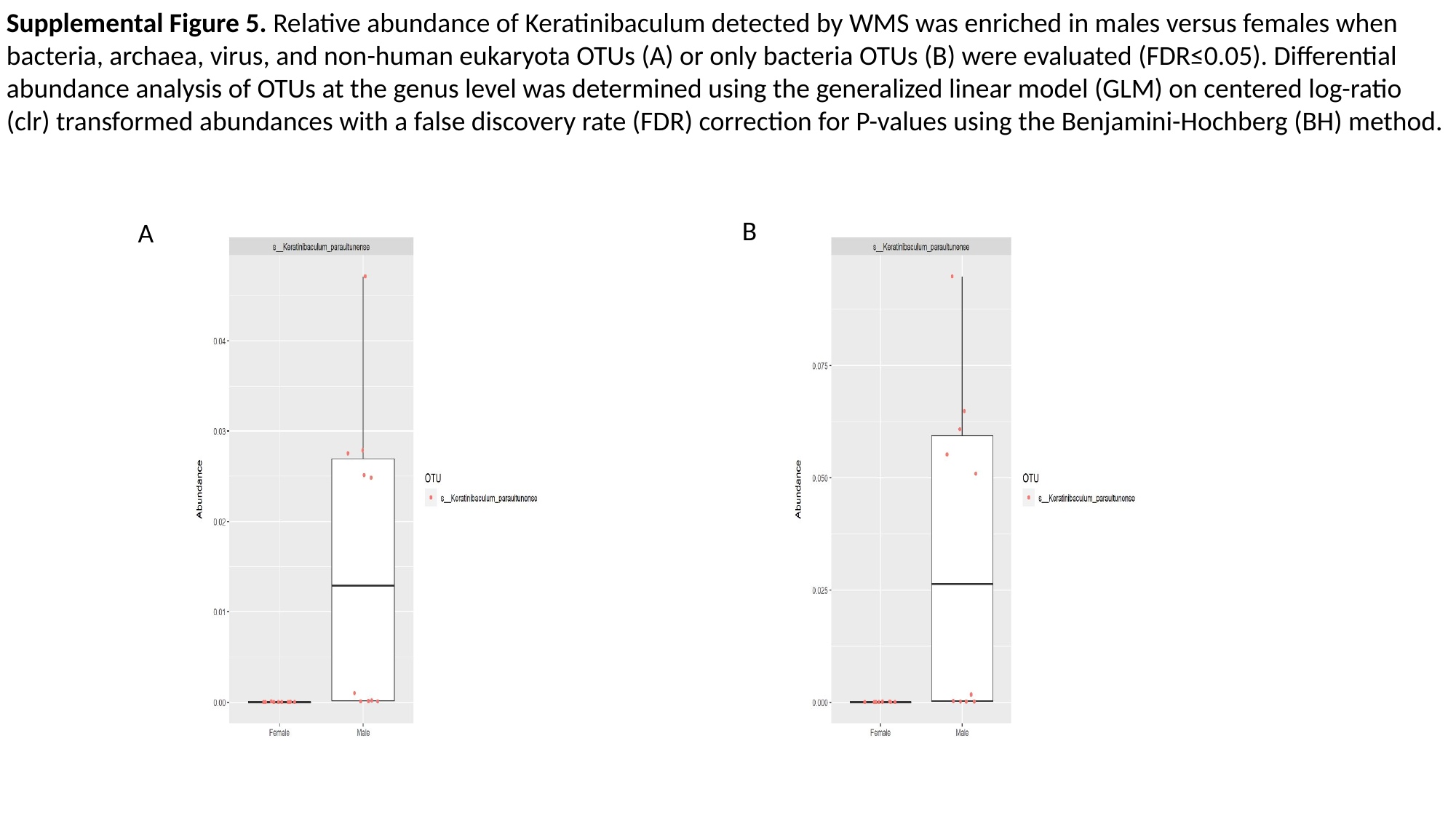

Supplemental Figure 5. Relative abundance of Keratinibaculum detected by WMS was enriched in males versus females when bacteria, archaea, virus, and non-human eukaryota OTUs (A) or only bacteria OTUs (B) were evaluated (FDR≤0.05). Differential abundance analysis of OTUs at the genus level was determined using the generalized linear model (GLM) on centered log-ratio (clr) transformed abundances with a false discovery rate (FDR) correction for P-values using the Benjamini-Hochberg (BH) method.
B
A

## Slide 9
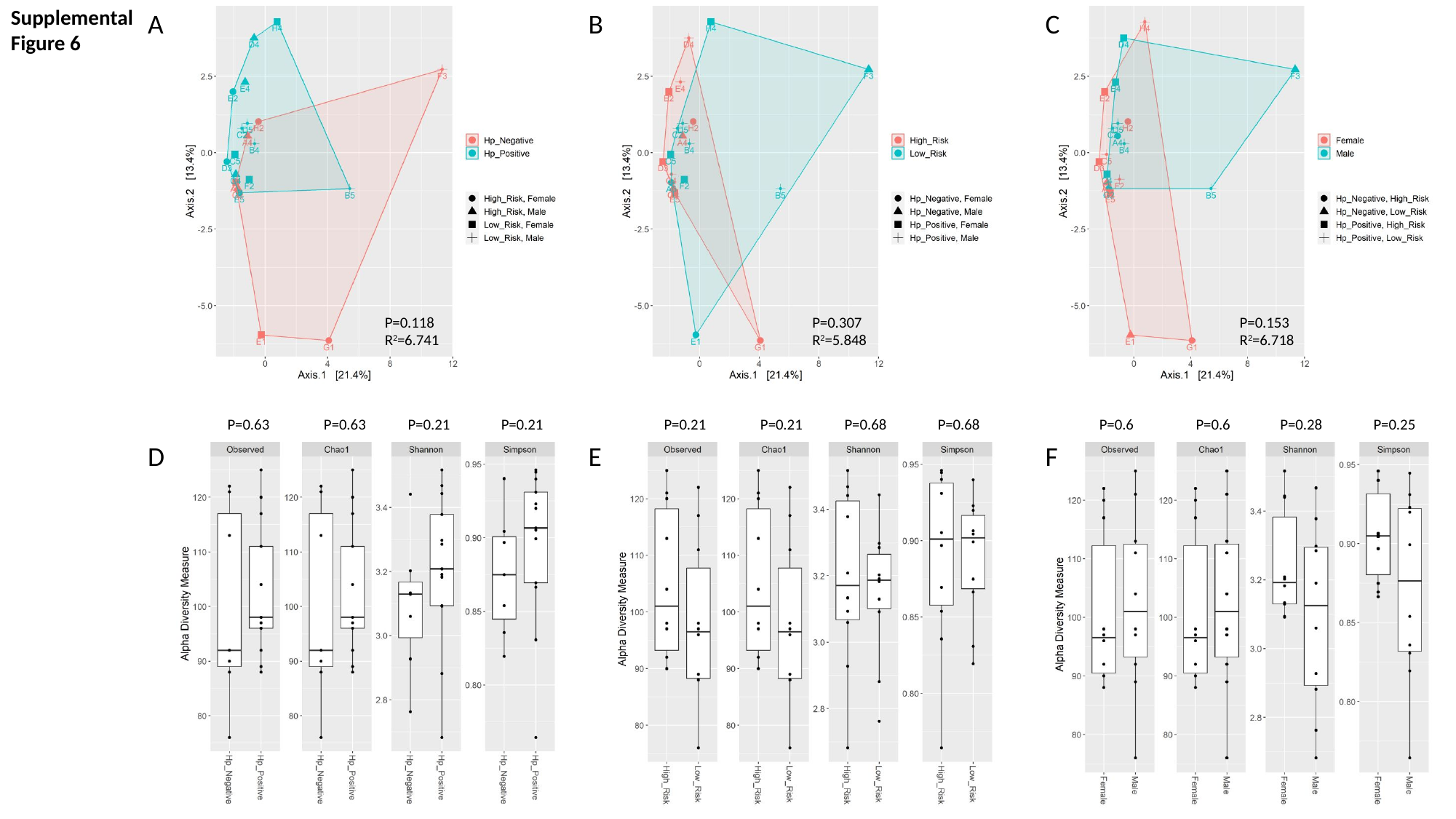

Supplemental Figure 6
A
B
C
P=0.118
R2=6.741
P=0.307
R2=5.848
P=0.153
R2=6.718
P=0.63
P=0.63
P=0.21
P=0.21
P=0.21
P=0.21
P=0.68
P=0.68
P=0.6
P=0.6
P=0.28
P=0.25
D
E
F

## Slide 10
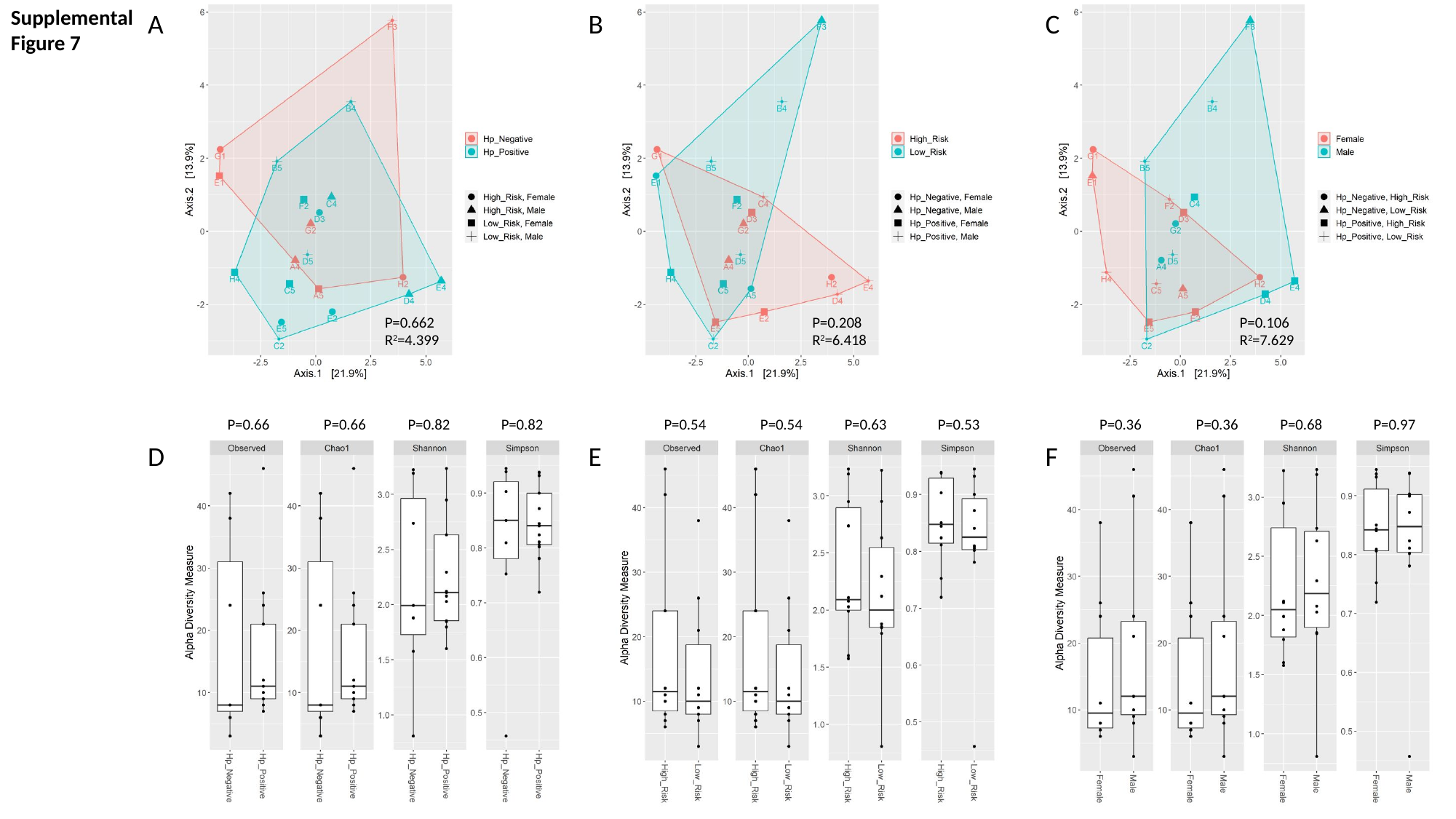

Supplemental Figure 7
A
B
C
P=0.662
R2=4.399
P=0.208
R2=6.418
P=0.106
R2=7.629
P=0.66
P=0.66
P=0.82
P=0.82
P=0.54
P=0.54
P=0.63
P=0.53
P=0.36
P=0.36
P=0.68
P=0.97
D
E
F

## Slide 11
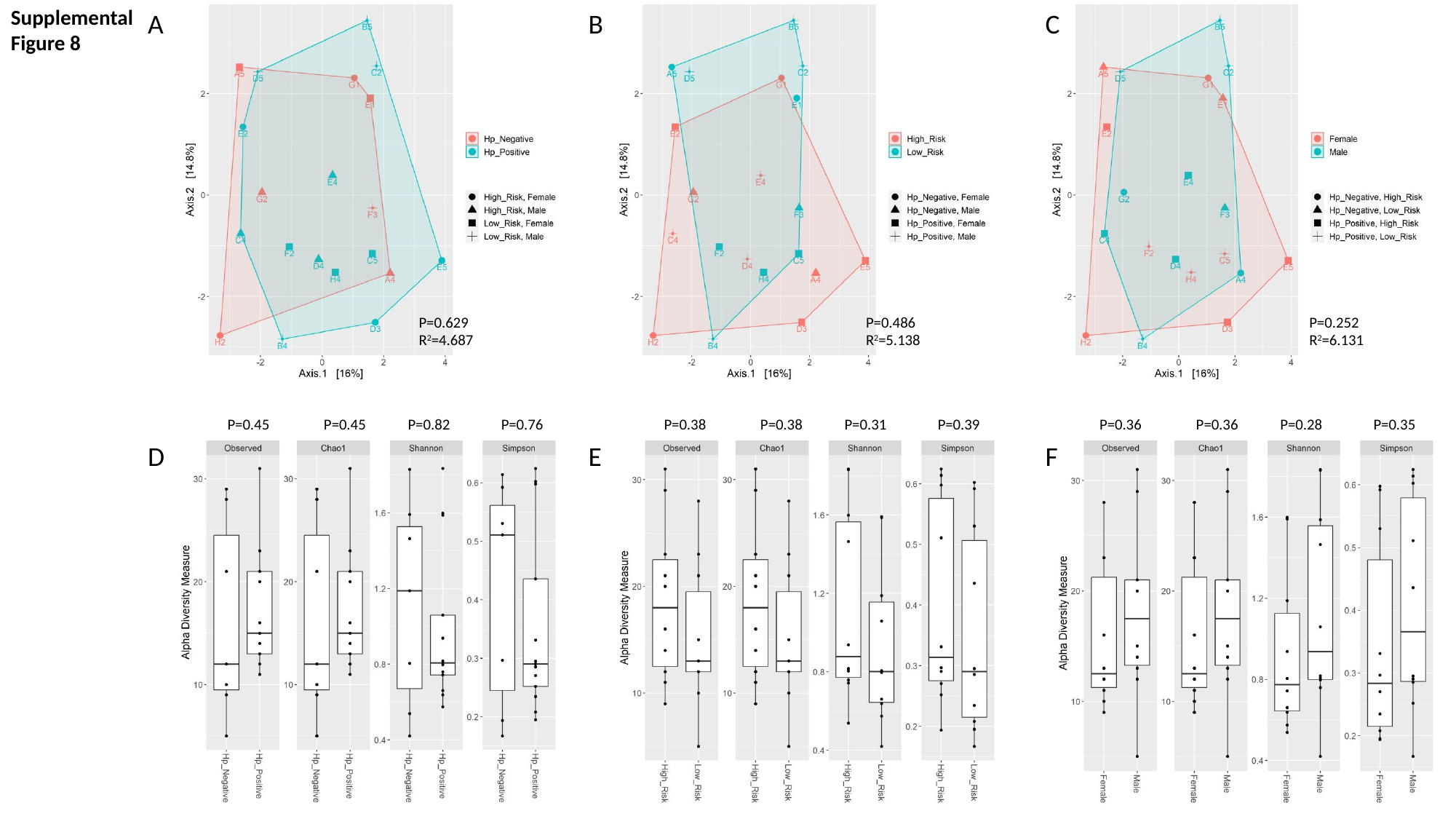

Supplemental Figure 8
A
B
C
P=0.629
R2=4.687
P=0.486
R2=5.138
P=0.252
R2=6.131
P=0.45
P=0.45
P=0.82
P=0.76
P=0.38
P=0.38
P=0.31
P=0.39
P=0.36
P=0.36
P=0.28
P=0.35
D
E
F

## Slide 12
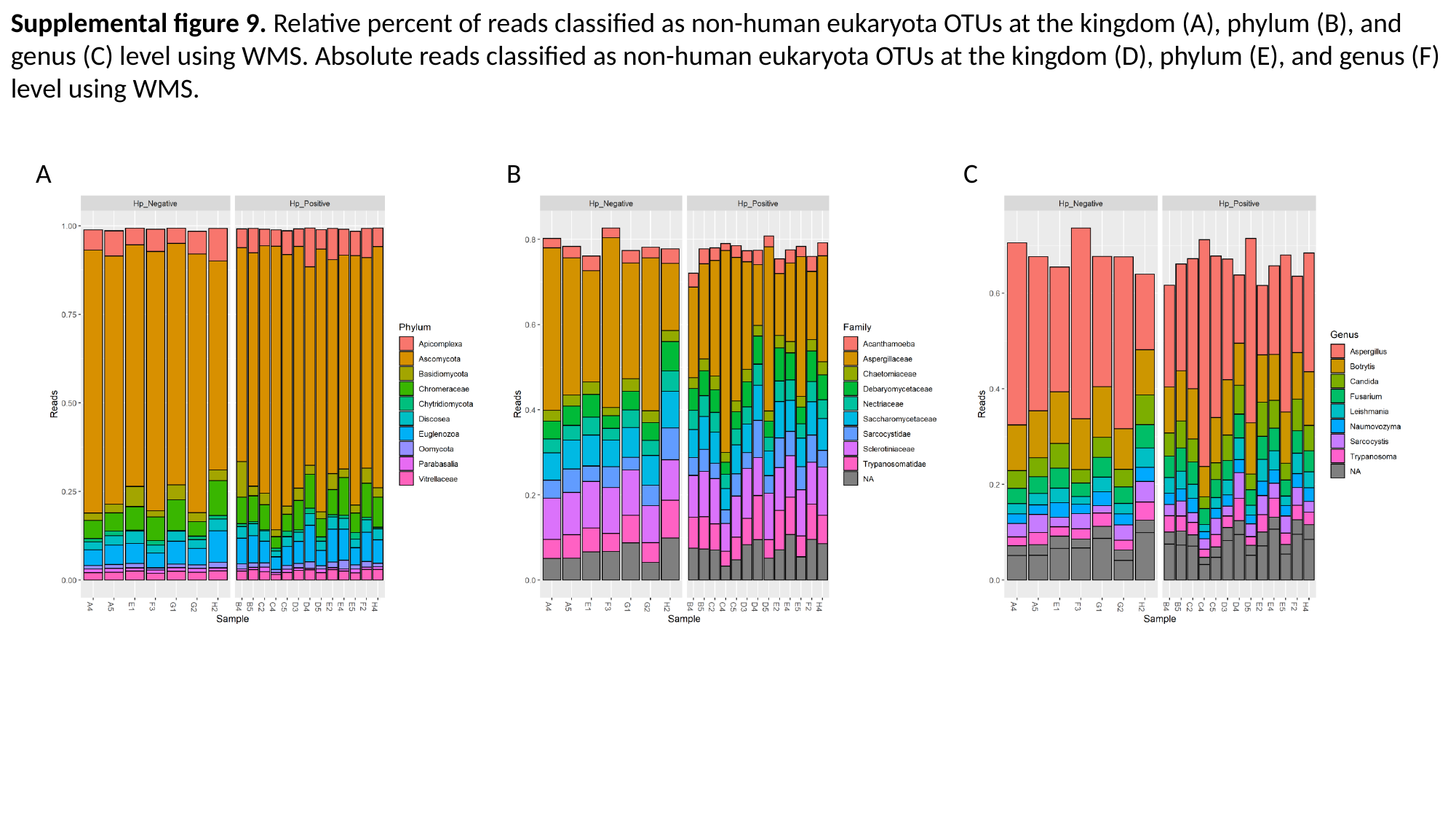

Supplemental figure 9. Relative percent of reads classified as non-human eukaryota OTUs at the kingdom (A), phylum (B), and genus (C) level using WMS. Absolute reads classified as non-human eukaryota OTUs at the kingdom (D), phylum (E), and genus (F) level using WMS.
A
B
C

## Slide 13
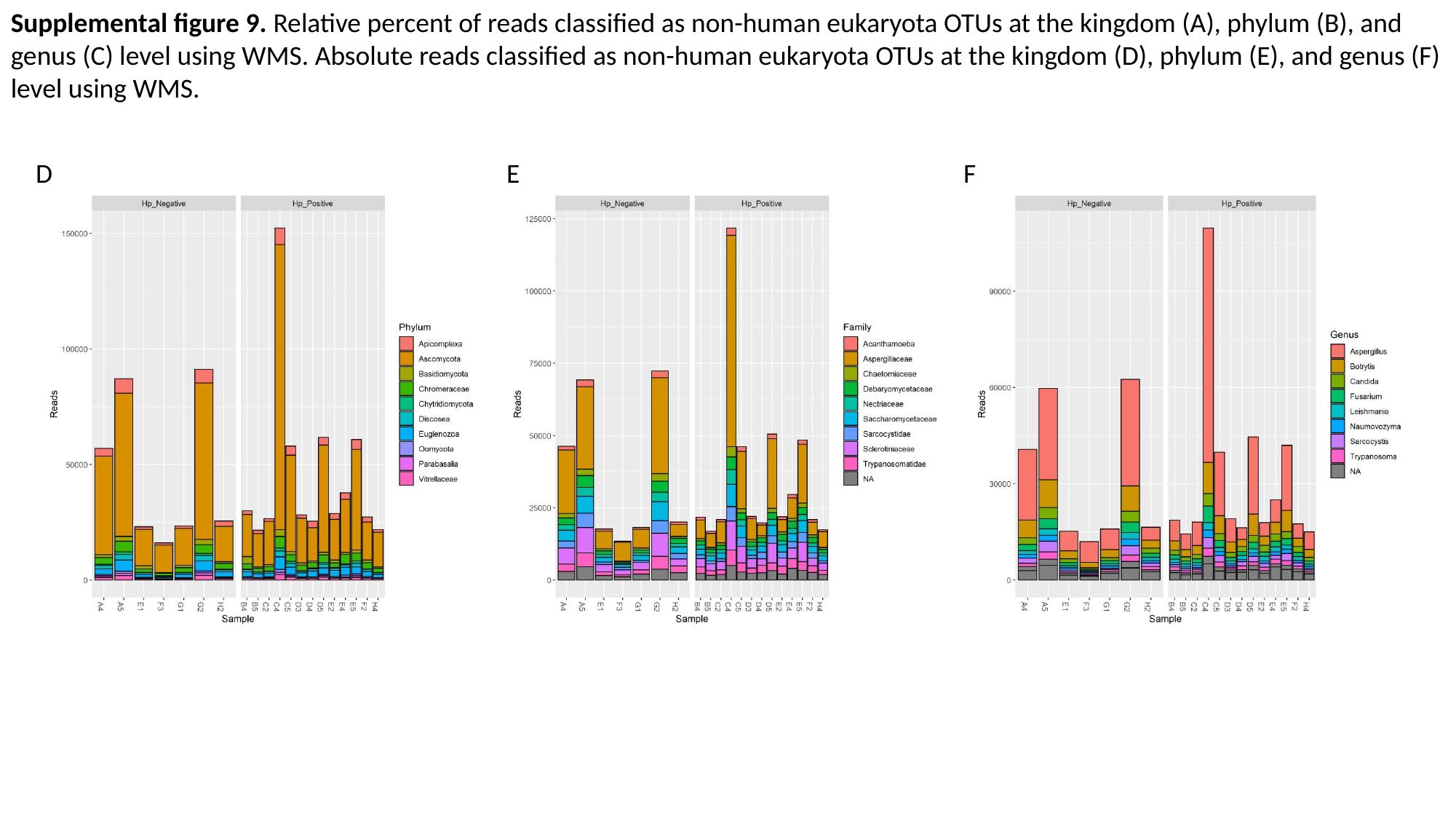

Supplemental figure 9. Relative percent of reads classified as non-human eukaryota OTUs at the kingdom (A), phylum (B), and genus (C) level using WMS. Absolute reads classified as non-human eukaryota OTUs at the kingdom (D), phylum (E), and genus (F) level using WMS.
D
E
F

## Slide 14
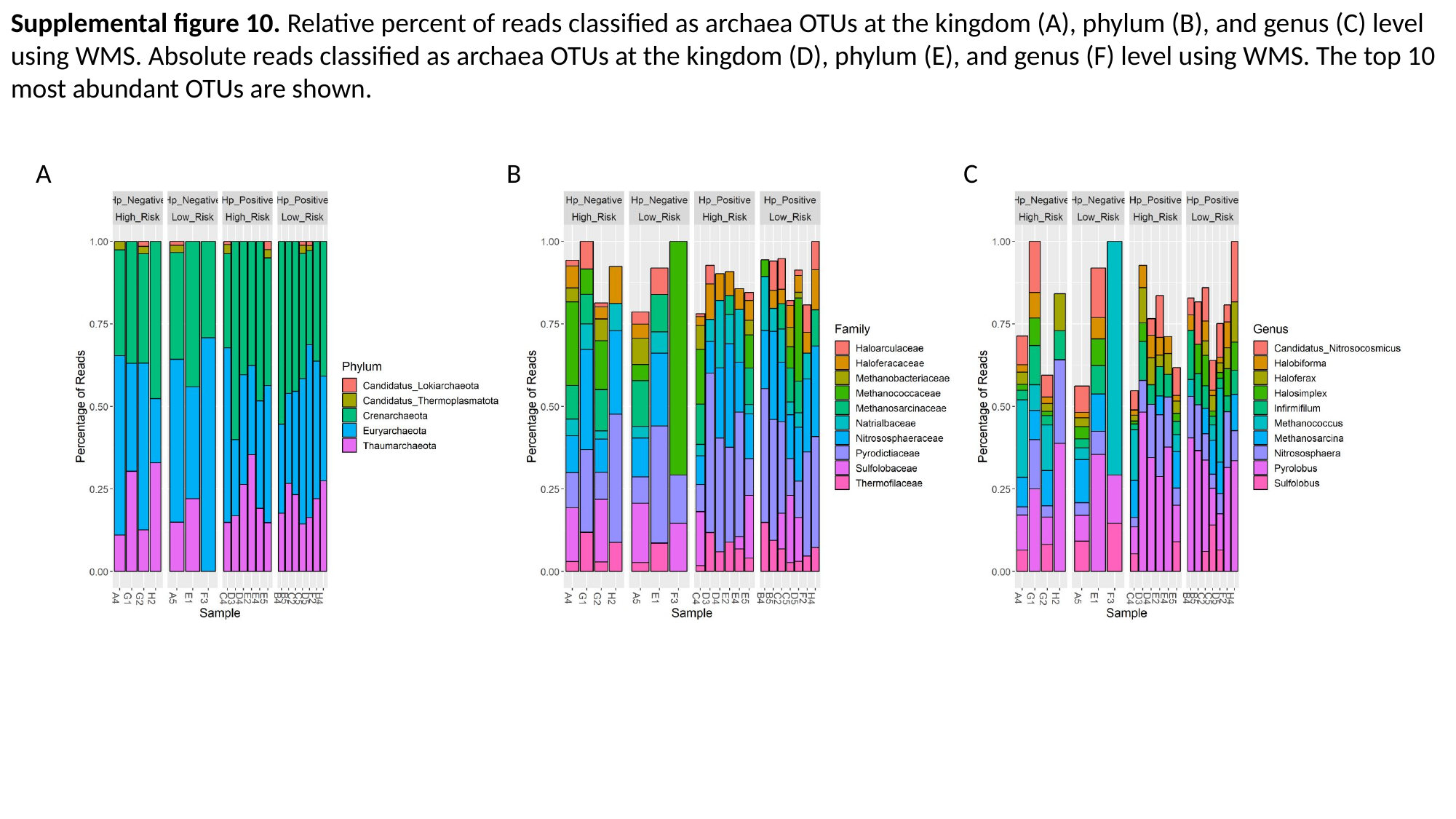

Supplemental figure 10. Relative percent of reads classified as archaea OTUs at the kingdom (A), phylum (B), and genus (C) level using WMS. Absolute reads classified as archaea OTUs at the kingdom (D), phylum (E), and genus (F) level using WMS. The top 10 most abundant OTUs are shown.
A
B
C

## Slide 15
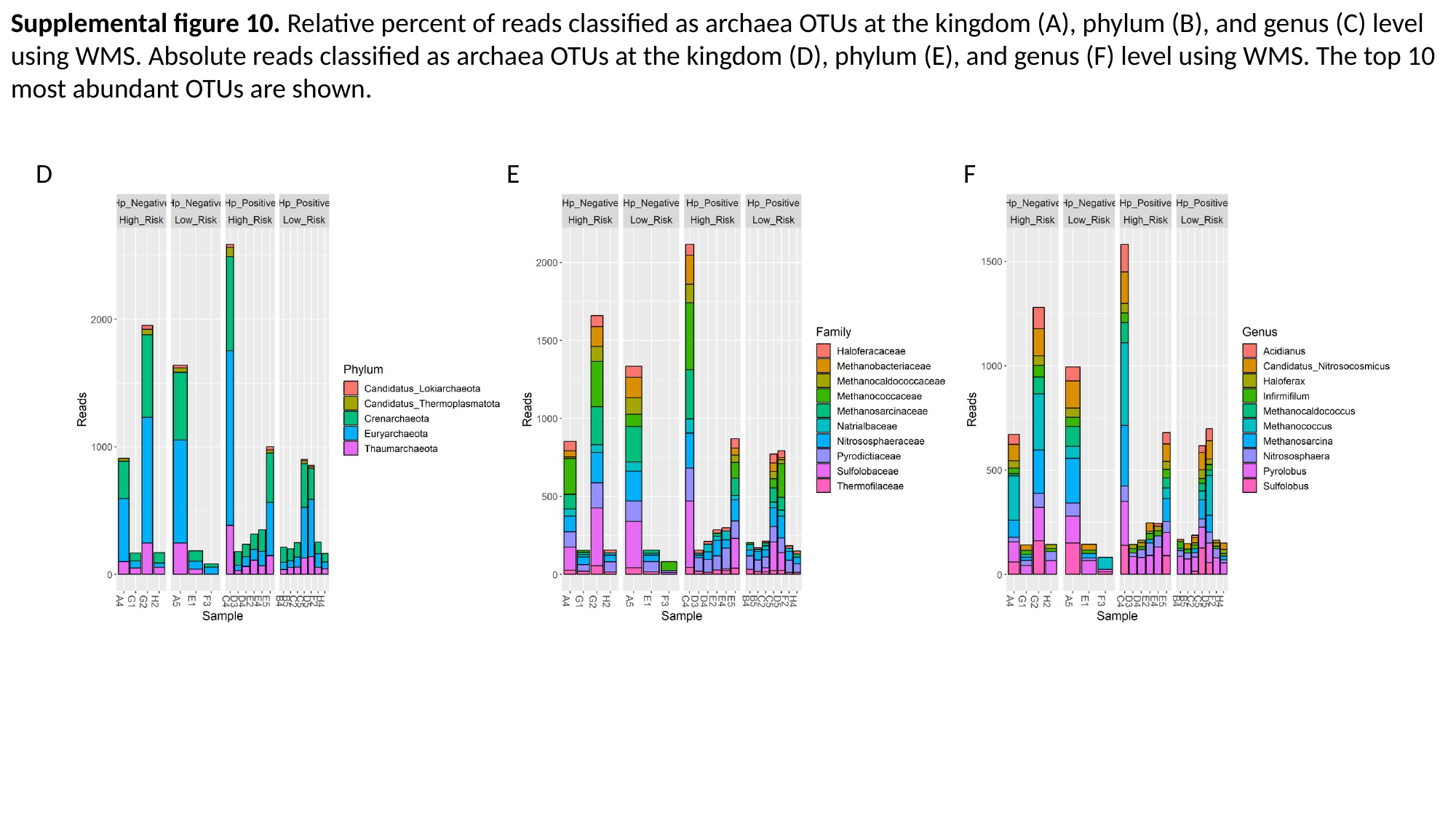

Supplemental figure 10. Relative percent of reads classified as archaea OTUs at the kingdom (A), phylum (B), and genus (C) level using WMS. Absolute reads classified as archaea OTUs at the kingdom (D), phylum (E), and genus (F) level using WMS. The top 10 most abundant OTUs are shown.
D
E
F

## Slide 16
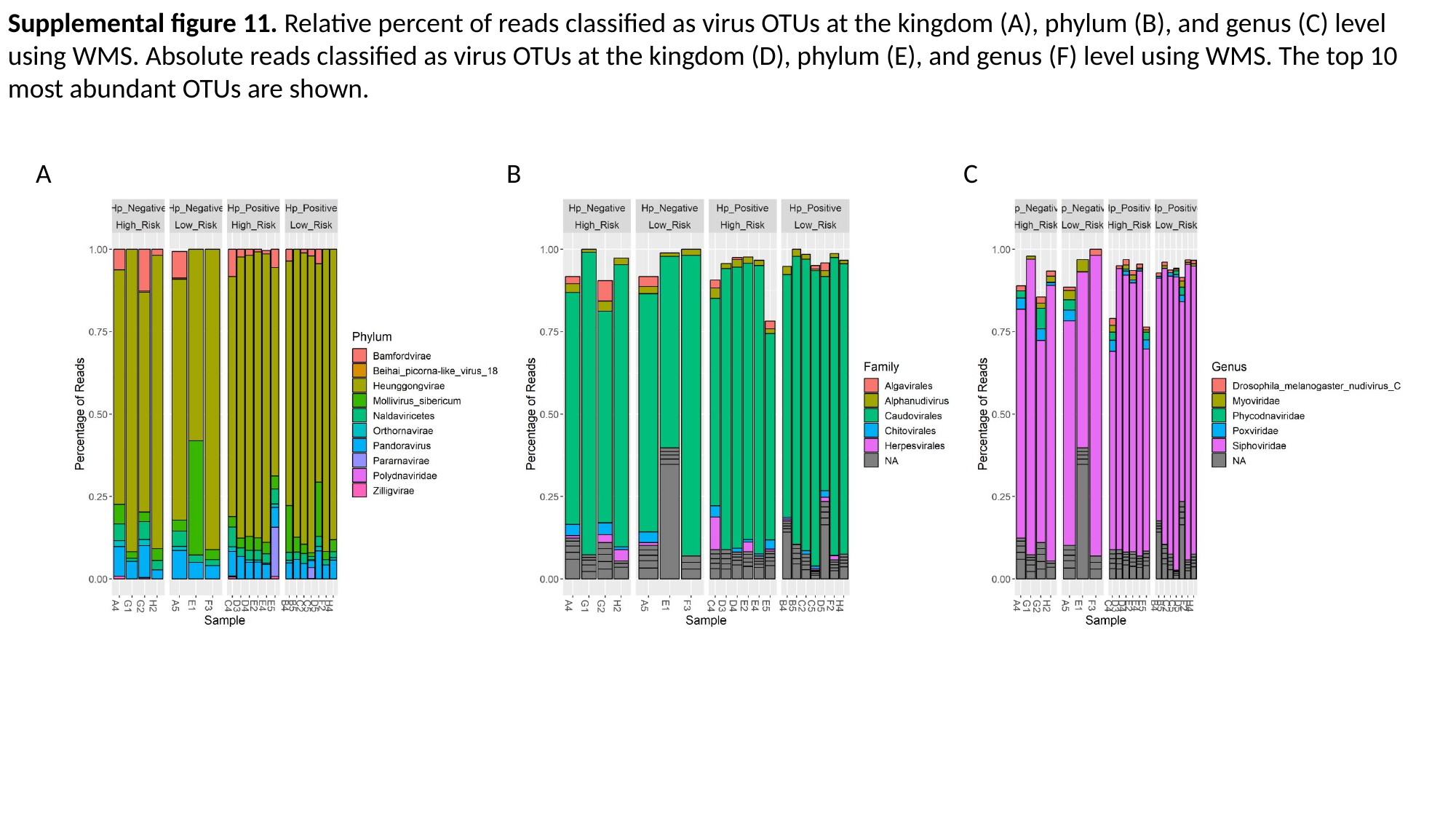

Supplemental figure 11. Relative percent of reads classified as virus OTUs at the kingdom (A), phylum (B), and genus (C) level using WMS. Absolute reads classified as virus OTUs at the kingdom (D), phylum (E), and genus (F) level using WMS. The top 10 most abundant OTUs are shown.
A
B
C

## Slide 17
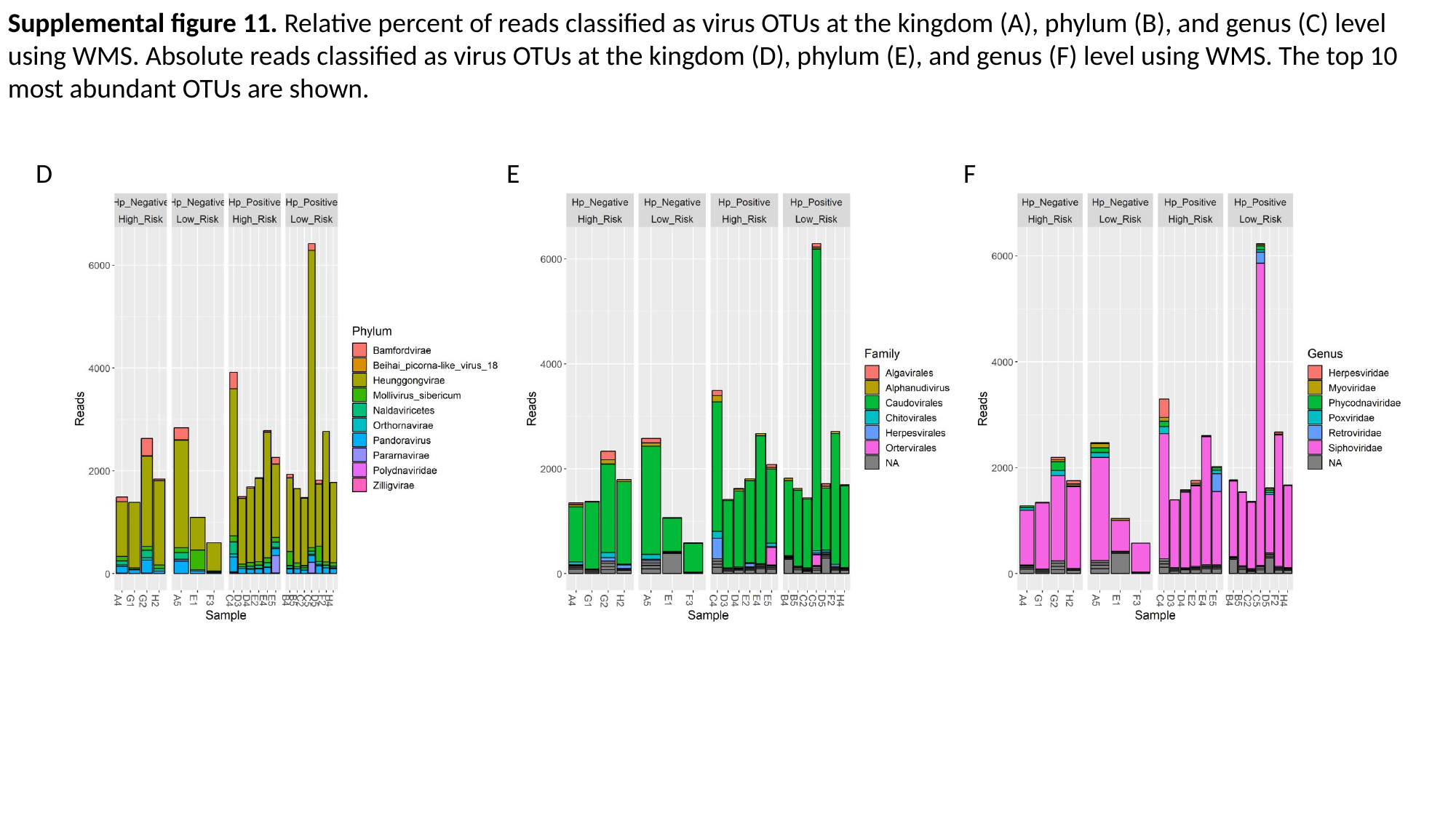

Supplemental figure 11. Relative percent of reads classified as virus OTUs at the kingdom (A), phylum (B), and genus (C) level using WMS. Absolute reads classified as virus OTUs at the kingdom (D), phylum (E), and genus (F) level using WMS. The top 10 most abundant OTUs are shown.
D
E
F

## Slide 18
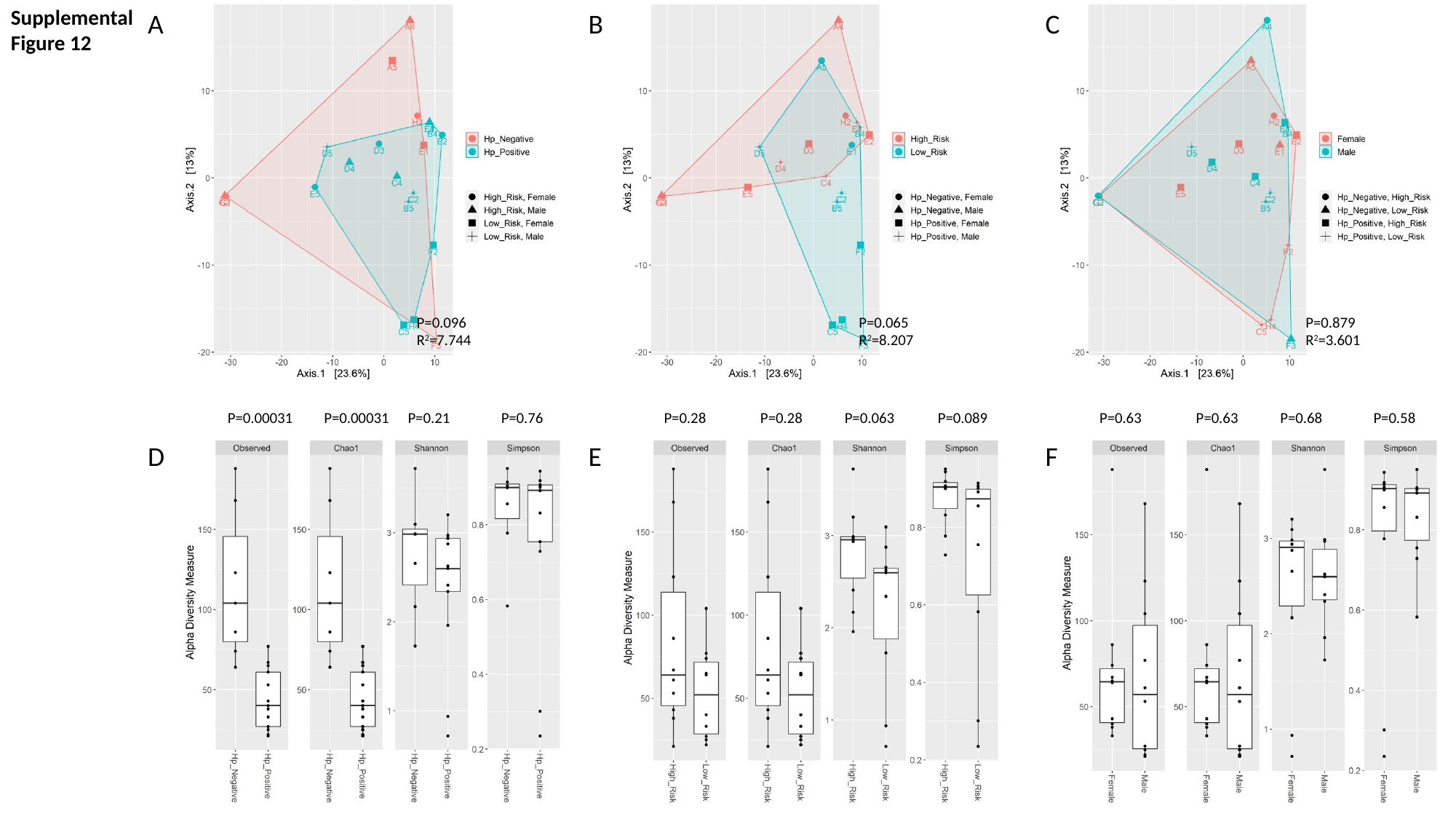

Supplemental Figure 12
A
B
C
P=0.096
R2=7.744
P=0.879
R2=3.601
P=0.065
R2=8.207
P=0.00031
P=0.00031
P=0.21
P=0.76
P=0.28
P=0.28
P=0.063
P=0.089
P=0.63
P=0.63
P=0.68
P=0.58
D
E
F

## Slide 19
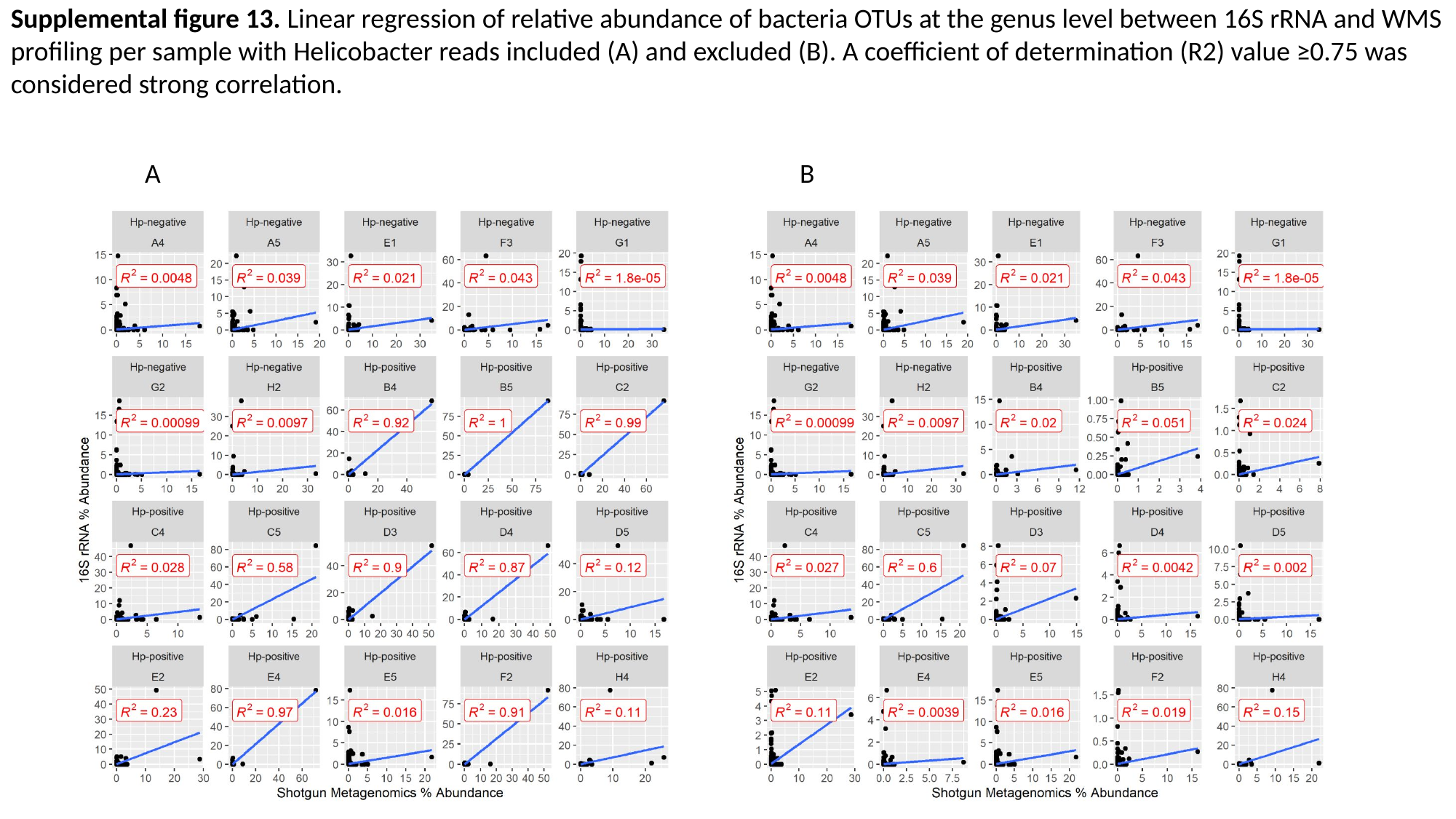

Supplemental figure 13. Linear regression of relative abundance of bacteria OTUs at the genus level between 16S rRNA and WMS profiling per sample with Helicobacter reads included (A) and excluded (B). A coefficient of determination (R2) value ≥0.75 was considered strong correlation.
A
B

## Slide 20
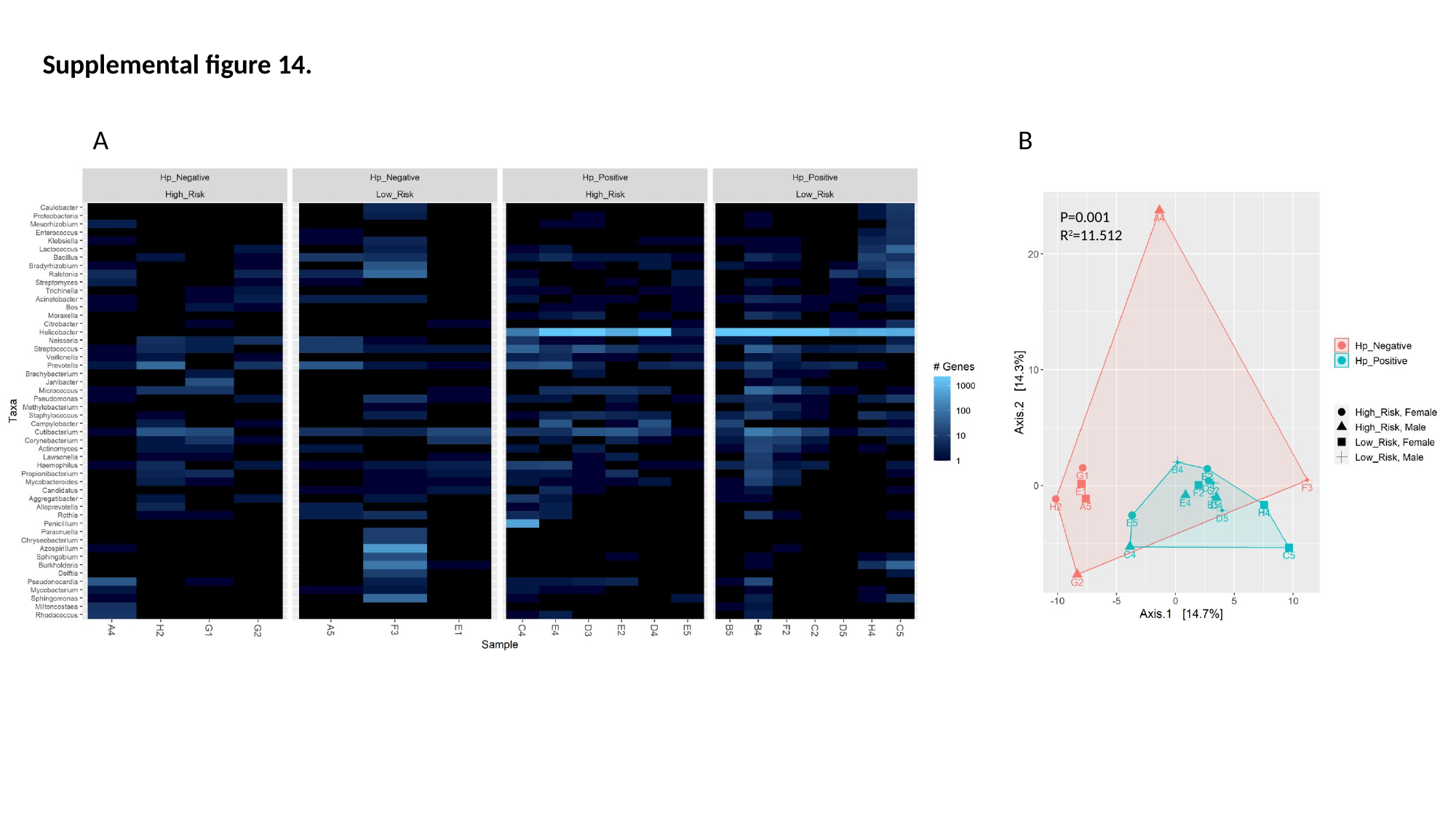

Supplemental figure 14.
A
B
P=0.001
R2=11.512

## Slide 21
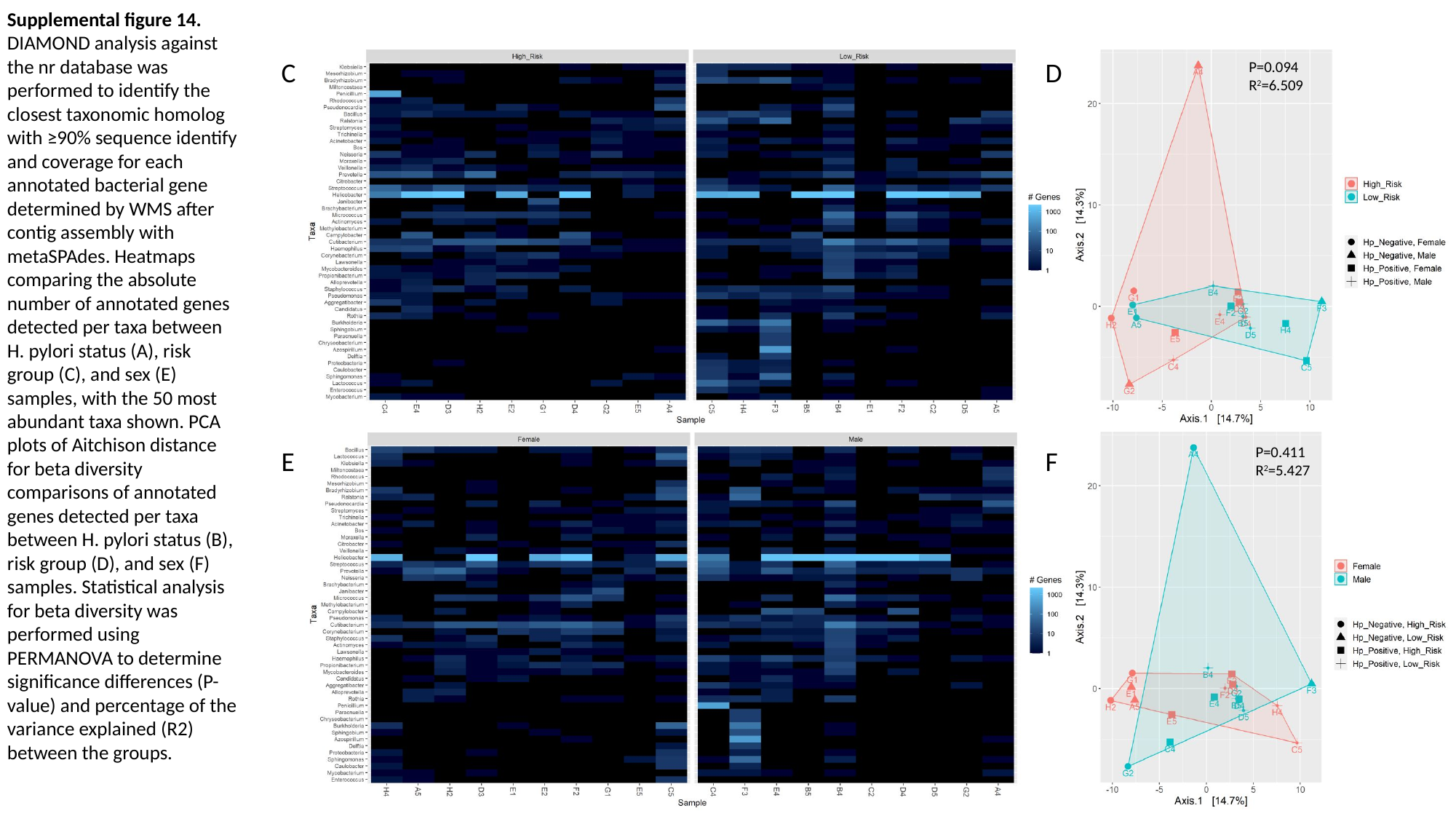

Supplemental figure 14. DIAMOND analysis against the nr database was performed to identify the closest taxonomic homolog with ≥90% sequence identify and coverage for each annotated bacterial gene determined by WMS after contig assembly with metaSPAdes. Heatmaps comparing the absolute number of annotated genes detected per taxa between H. pylori status (A), risk group (C), and sex (E) samples, with the 50 most abundant taxa shown. PCA plots of Aitchison distance for beta diversity comparisons of annotated genes detected per taxa between H. pylori status (B), risk group (D), and sex (F) samples. Statistical analysis for beta diversity was performed using PERMANOVA to determine significance differences (P-value) and percentage of the variance explained (R2) between the groups.
C
D
P=0.094
R2=6.509
P=0.411
R2=5.427
E
F

## Slide 22
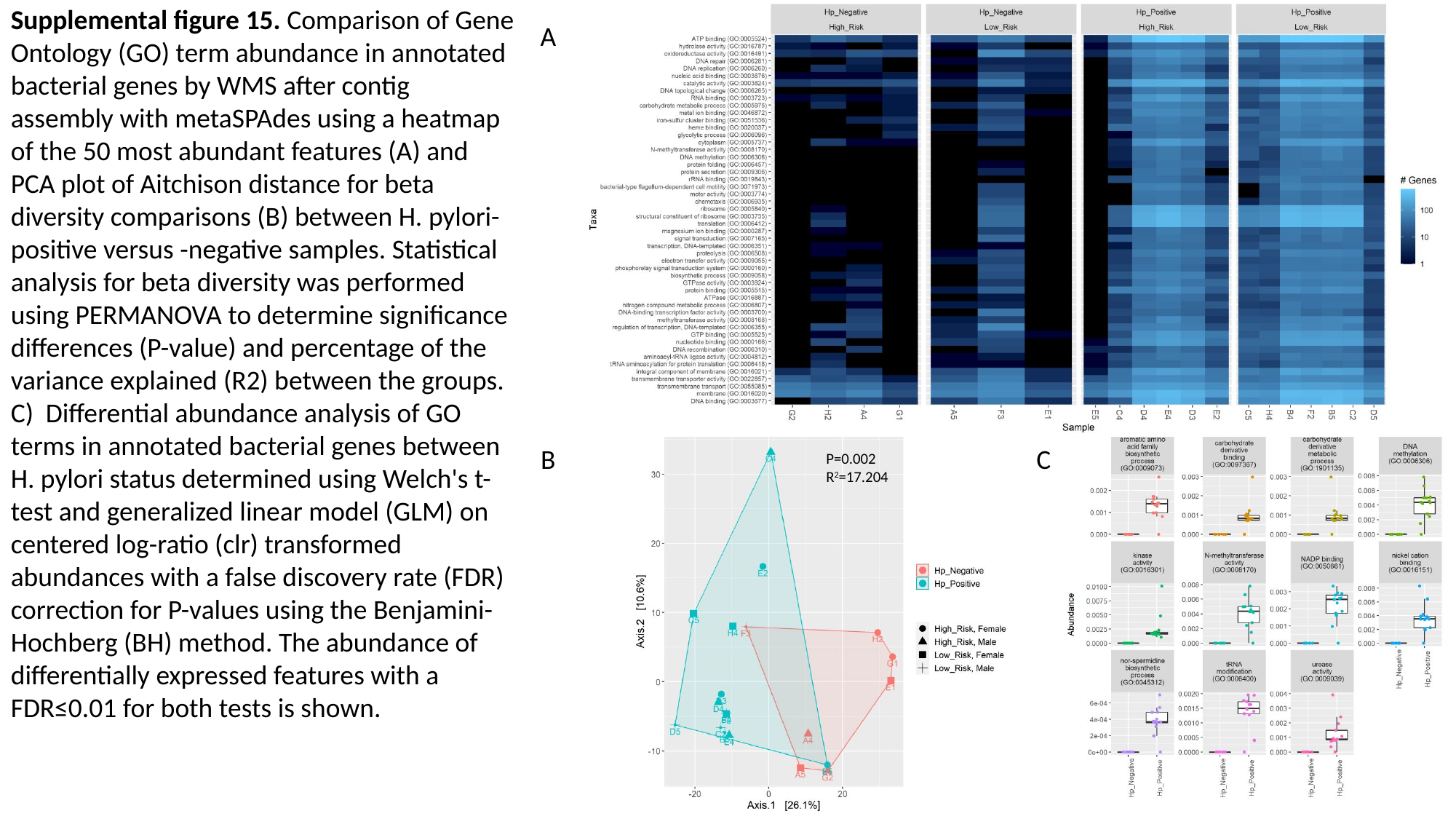

Supplemental figure 15. Comparison of Gene Ontology (GO) term abundance in annotated bacterial genes by WMS after contig assembly with metaSPAdes using a heatmap of the 50 most abundant features (A) and PCA plot of Aitchison distance for beta diversity comparisons (B) between H. pylori-positive versus -negative samples. Statistical analysis for beta diversity was performed using PERMANOVA to determine significance differences (P-value) and percentage of the variance explained (R2) between the groups. C) Differential abundance analysis of GO terms in annotated bacterial genes between H. pylori status determined using Welch's t-test and generalized linear model (GLM) on centered log-ratio (clr) transformed abundances with a false discovery rate (FDR) correction for P-values using the Benjamini-Hochberg (BH) method. The abundance of differentially expressed features with a FDR≤0.01 for both tests is shown.
A
B
C
P=0.002
R2=17.204

## Slide 23
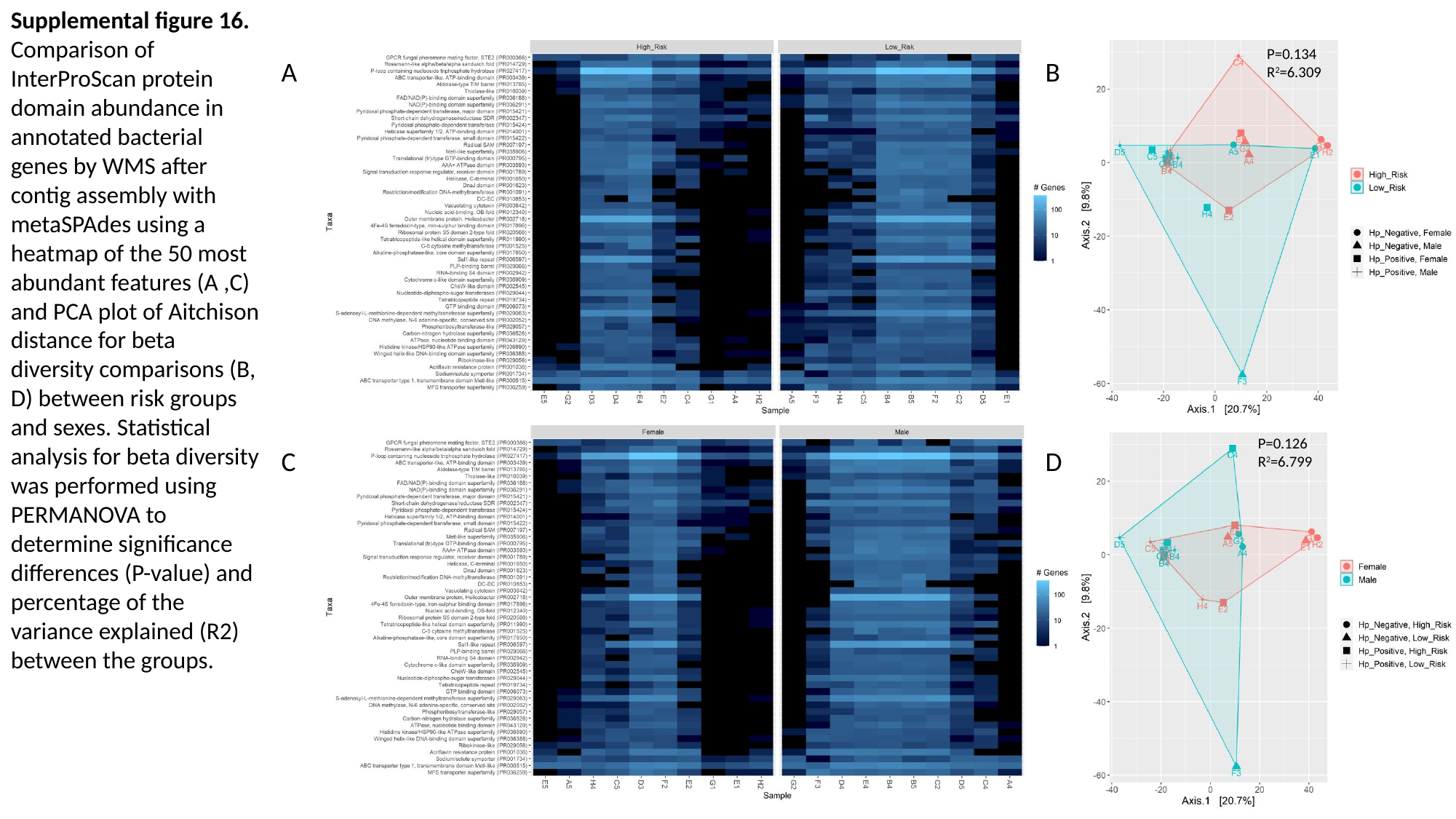

Supplemental figure 16. Comparison of InterProScan protein domain abundance in annotated bacterial genes by WMS after contig assembly with metaSPAdes using a heatmap of the 50 most abundant features (A ,C) and PCA plot of Aitchison distance for beta diversity comparisons (B, D) between risk groups and sexes. Statistical analysis for beta diversity was performed using PERMANOVA to determine significance differences (P-value) and percentage of the variance explained (R2) between the groups.
P=0.134
R2=6.309
A
B
P=0.126
R2=6.799
C
D

## Slide 24
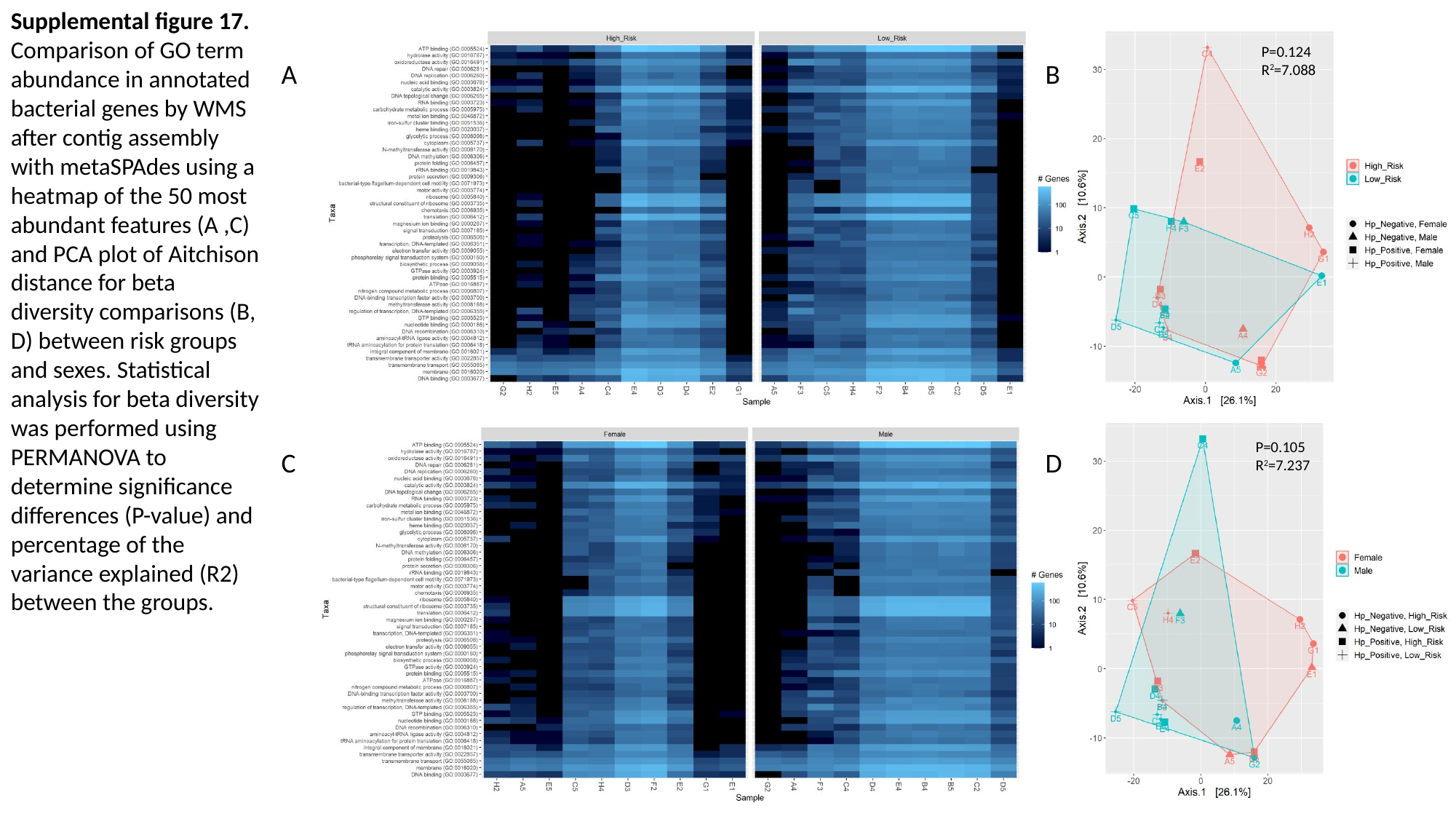

Supplemental figure 17. Comparison of GO term abundance in annotated bacterial genes by WMS after contig assembly with metaSPAdes using a heatmap of the 50 most abundant features (A ,C) and PCA plot of Aitchison distance for beta diversity comparisons (B, D) between risk groups and sexes. Statistical analysis for beta diversity was performed using PERMANOVA to determine significance differences (P-value) and percentage of the variance explained (R2) between the groups.
P=0.124
R2=7.088
A
B
P=0.105
R2=7.237
C
D

## Slide 25
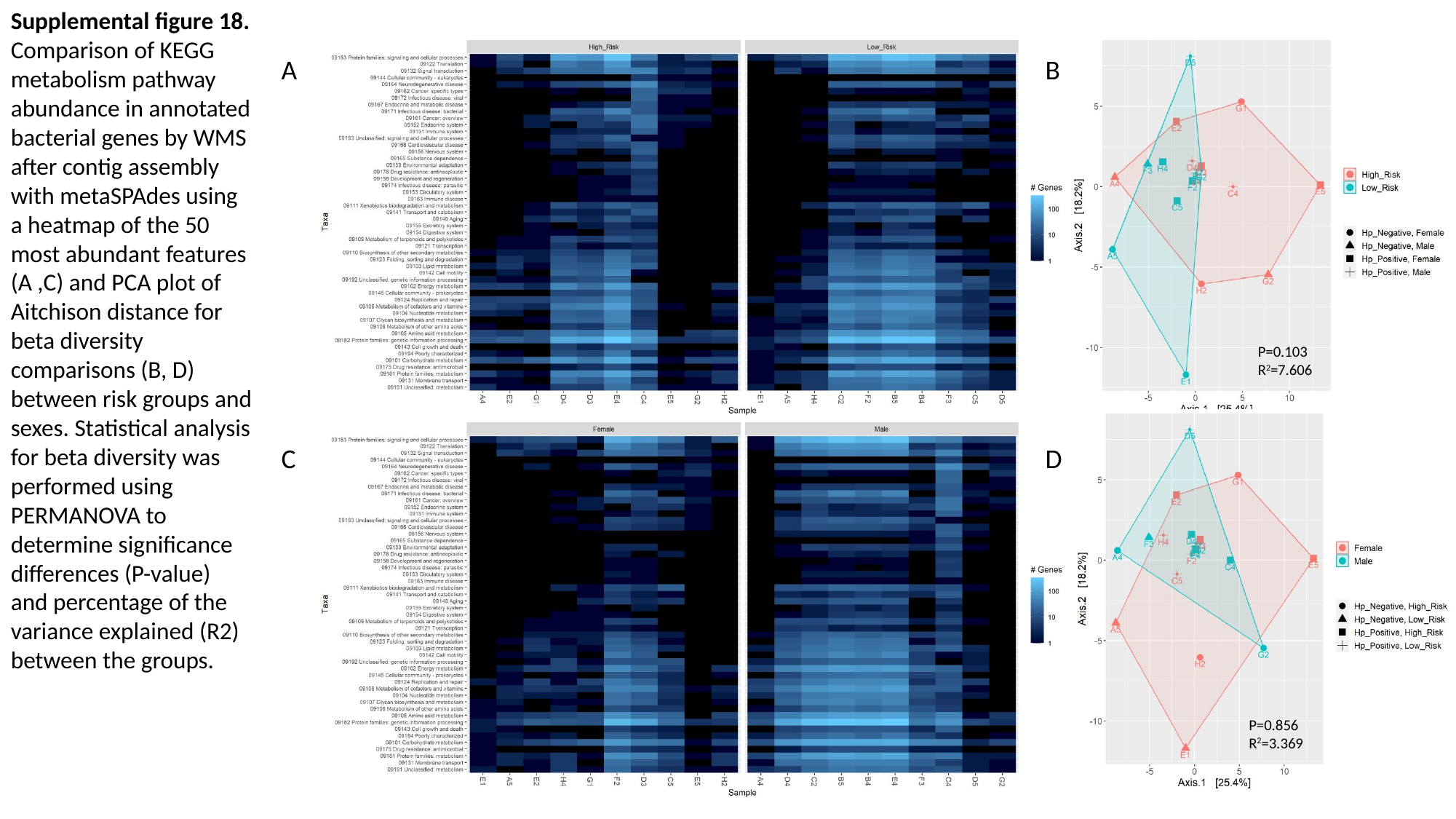

Supplemental figure 18. Comparison of KEGG metabolism pathway abundance in annotated bacterial genes by WMS after contig assembly with metaSPAdes using a heatmap of the 50 most abundant features (A ,C) and PCA plot of Aitchison distance for beta diversity comparisons (B, D) between risk groups and sexes. Statistical analysis for beta diversity was performed using PERMANOVA to determine significance differences (P-value) and percentage of the variance explained (R2) between the groups.
A
B
P=0.103
R2=7.606
C
D
P=0.856
R2=3.369

## Slide 26
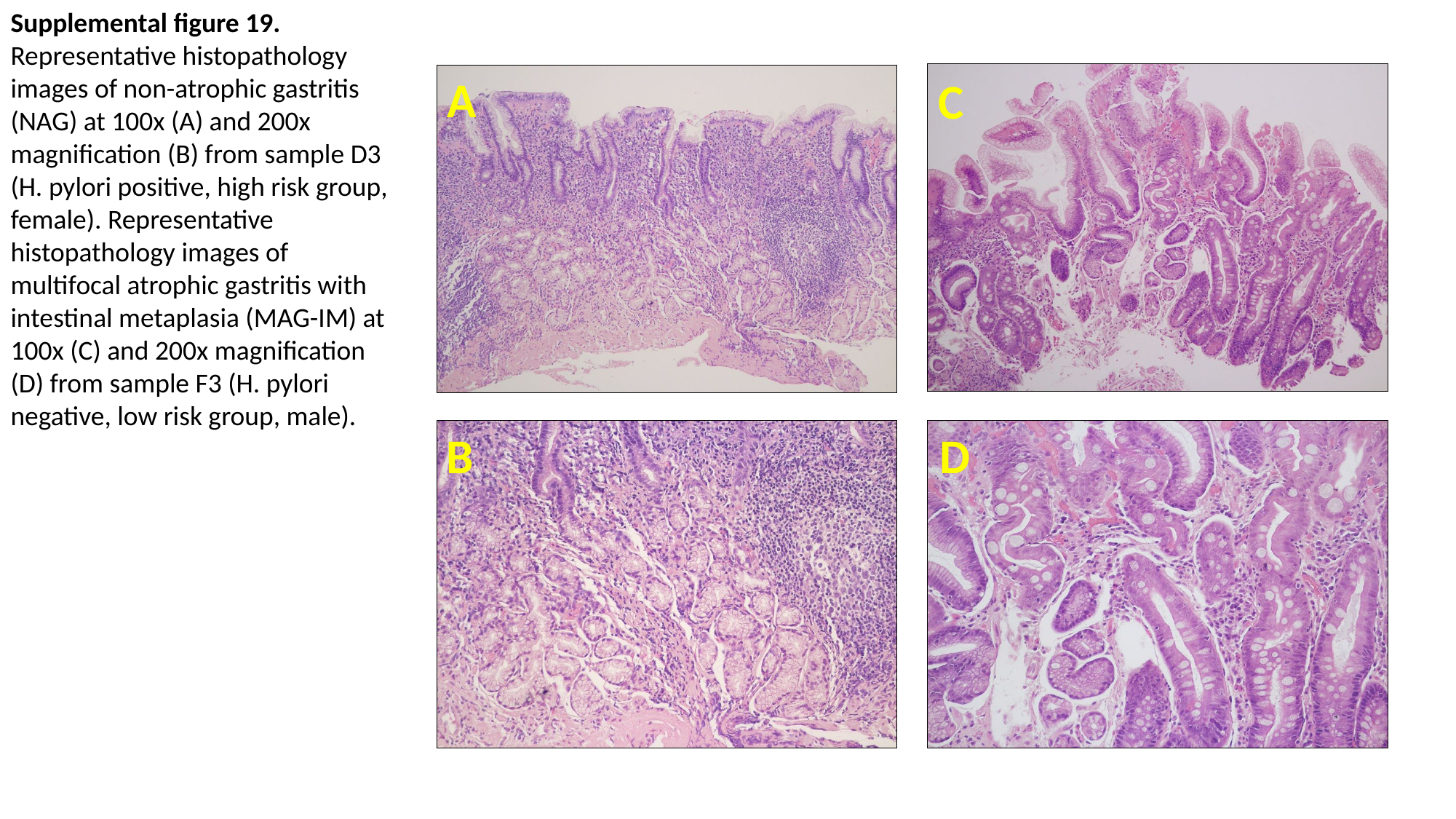

Supplemental figure 19. Representative histopathology images of non-atrophic gastritis (NAG) at 100x (A) and 200x magnification (B) from sample D3 (H. pylori positive, high risk group, female). Representative histopathology images of multifocal atrophic gastritis with intestinal metaplasia (MAG-IM) at 100x (C) and 200x magnification (D) from sample F3 (H. pylori negative, low risk group, male).
A
C
D
B
